# Cytotoxic Stilbenoids, Hetero- and Homodimers of Homoisoflavonoids from *Prospero autumnale*

**DOI:** 10.1021/acs.jnatprod.4c01263

**Published:** 2025-01-24

**Authors:** Hasan Kırmızıbekmez, Başak Aru, Jana Křoustková, Murat Erdoğan, Ian Torrence, Kaori Ando, Dean J. Tantillo, Milan Malaník, Štefan Kosturko, Jiří Kuneš, Lucie Cahlíková

**Affiliations:** † Department of Pharmacognosy, Faculty of Pharmacy, Yeditepe University, TR-34755 Kayışdağı, İstanbul, Türkiye; ‡ Department of Immunology, Faculty of Medicine, Yeditepe University, TR-34755 Kayışdağı, İstanbul, Türkiye; § Department of Pharmacognosy and Pharmaceutical Botany, Faculty of Pharmacy, Charles University, Heyrovského 1203, 500 03 Hradec Kralove, Czech Republic; ∥ Department of Chemistry, University of California, Davis, California 95616, United States; ⊥ Department of Natural Drugs, Faculty of Pharmacy, Masaryk University, Palackého třída 1946/1, 61200 Brno, Czech Republic; # Department of Organic and Bioorganic Chemistry, Faculty of Pharmacy, Charles University, Heyrovského 1203, 500 03 Hradec Kralove, Czech Republic; ∇ Department of Analytical Chemistry, Faculty of Pharmacy, Charles University, Heyrovského 1203, 500 03 Hradec Kralove, Czech Republic

## Abstract

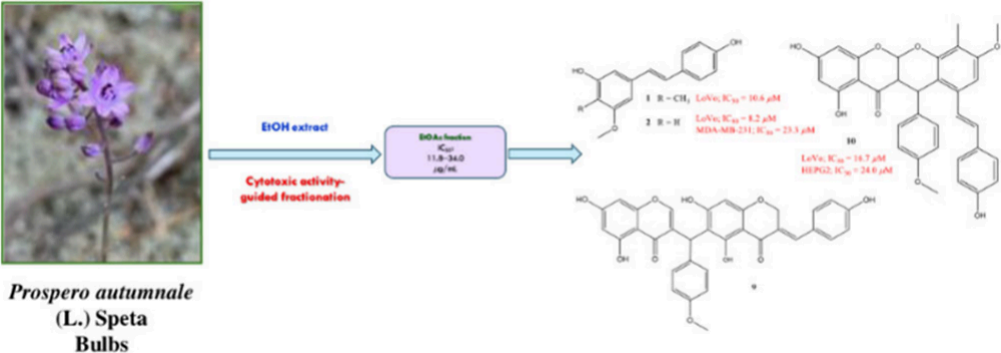

An activity-guided isolation study on the EtOH extract prepared from the bulbs of *Prospero autumnale* yielded four new phenolic compounds, including a new stilbenoid (**1**), a new homoisoflavonoid derivative (**8**), a new homoisoflavonoid dimer (**9**), and an unprecedented homoisoflavone–stilbene heterodimer (**10**), together with six known (**2**–**7**) analogs. Their chemical structures were elucidated by spectroscopic analysis and theoretical NMR and ECD calculations. Compounds **9** and **10** are unique in their scaffolds. The *in vitro* cytotoxic activity of purified compounds was evaluated against eight tumor cell lines (HCT116, LoVo, DU145, PC3, HEP3B, HEPG2, MCF7, and MDA-MB-231) and one nontumor cell line (L929) by the MTS assay. Compounds **1**, **2**, **4**, and **10** exhibited inhibition with IC_50_ values ranging from 8.2 to 37.6 μM. Cytotoxic cell death mechanisms were further investigated, indicating variability in apoptosis, necrosis, or cell cycle arrest.

*Prospero* Salisb. is a plant genus mainly distributed in the Mediterranean region, Europe, and the Middle East. It includes 17 accepted species according to the Plants of the World Online (POWO) and the World Flora Online (WFO) databases.^1,2^ The species *Prospero autumnale* (L.) Speta is a perennial bulbous herb belonging to the Asparagaceae family. There has been no report on the medicinal use of this species. Only one study described the HPLC-DAD detection of several phenolic compounds, mentioning the antioxidant and cytotoxic activities of extracts prepared from its underground and aerial parts.^3^ Homoisoflavonoids and stilbenoids have been previously isolated from Asparagaceae,^4,5^ but no previous isolation and characterization of natural products from *P. autumnale* has been reported. Homoisoflavonoids have limited distribution in the plant kingdom, mainly occurring in some genera, such as Asparagaceae, Fabaceae, Polygonaceae, Gentianaceae, Portulacaceae, and Orchidaceae. Homoisoflavonoids display various pharmacological activities, including cytotoxic, immunomodulatory, anti-inflammatory, antioxidant, antimicrobial, antidiabetic, vasorelaxant, and anticholinesterase effects.^6,7^ Stilbenoids, also polyphenolic compounds, present promising bioactivities such as anticancer, antiangiogenic, cardioprotective, antidiabetic, anti-inflammatory, and neuroprotective activities.^8^ The aim of this study was to isolate cytotoxic compounds from the EtOH extract prepared from the bulbs of *P. autumnale* through *in vitro* activity-guided fractionation.

## Results and Discussion

Bulbs (210 g) were extracted with EtOH. The extract was evaluated for its *in vitro* cytotoxic activity against the colon (HCT116 and LoVo), prostate (DU145 and PC3), liver (HEP3B and HEPG2), and breast (MCF7 and MDA-MB-231) cancer cells by MTS assay, displaying cytotoxic activity (IC_50_ = 15.1–86.1 μg/mL) against all cancer cell lines except for MDA-MB-231 (Table S1). Then, the EtOH extract was suspended in H_2_O and partitioned with EtOAc and *n*-BuOH. The EtOAc, *n*-BuOH, and H_2_O fractions were evaluated against the same panel of cell lines. Only the EtOAc fraction exhibited cytotoxicity against all of the tested cancer cell lines. Thus, the EtOAc fraction was subjected to chromatographic separations over SiO_2_, Sephadex LH-20, and MPLC, to yield a new stilbenoid (**1**), two new homoisoflavonoid derivatives (**8** and **9**), and a new homoisoflavone-stilbene heterodimer (**10**), along with six known compounds.

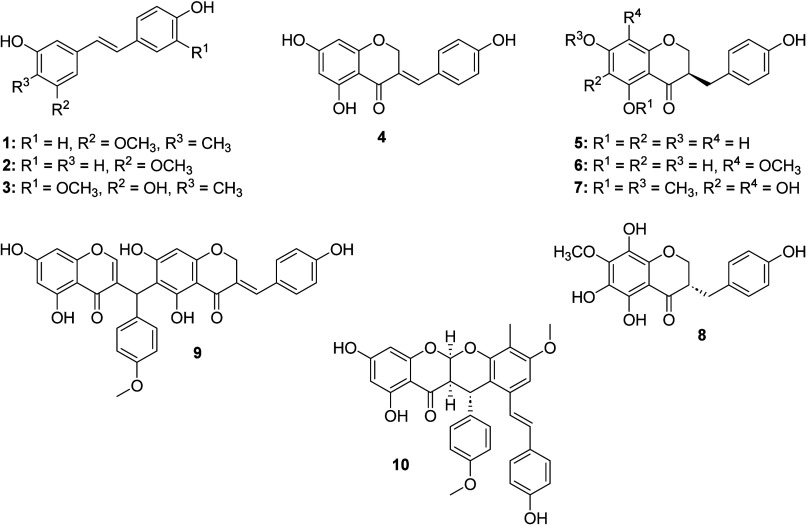



**Figure 1:**
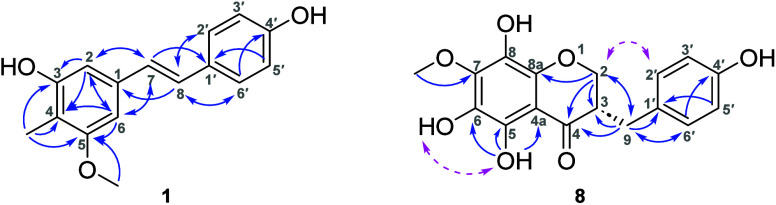
Key HMBC correlations (blue arrows) are shown for compounds **1** and **8**. Key ROESY correlations (pink dashed arrows) are for **8**.

Compound **1** was obtained as a pale amorphous powder. The HRESIMS revealed a protonated peak at *m*/*z* 257.1177 [M + H]^+^ (calcd. for C_16_H_17_O_3_^+^, 257.1172), indicating nine degrees of unsaturation. The ^1^H NMR spectrum of **1** exhibited two characteristic signals of AA′BB′ type aromatic protons (δ_H_ 7.38–7.34, m and 6.79–6.75, m), two *trans*-coupled olefinic protons with a *J*-coupling of 16.2 Hz (δ_H_ 6.98 and 6.85), and two doublets with a *J*-coupling of 1.5 Hz (δ_H_ 6.61 and 6.59), as well as a deshielded singlet of a methoxy group (δ_H_ 3.84, s) and a singlet of a typical methyl group (δ_H_ 2.04, s). The ^13^C NMR spectrum of **1** contained 16 resonances. All ^1^H and ^13^C signals were assigned to CH_3_/CH/C groups according to the cross-peaks in the HSQC experiment (Table 1). A stilbenoid scaffold was established by correlations from the HMBC experiment. The exact locations of the C-4 methyl were revealed by several long-range correlations between δ_H_ 2.04 and δ_C_ 157.1 (C-3), δ_C_ 112.9 (C-4), and δ_C_ 160.2 (C-5). Similarly, the methoxy group showed a long-range correlation with C-5. All of the key correlations from the HMBC experiment are depicted in Figure 1. Based on these data, the chemical structure of **1** was identified as 3,4′-dihydroxy-4-methyl-5-methoxystilbene and named prospestilbene.

**Table 1:** ^1^H (500 MHz) and ^13^C (125.7 MHz) NMR Data of **1** and **8**

	**1**^a^		**8**^b^	
no.	δ_C_, type	δ_H_, mult. (*J* in Hz)	δ_C_, type	δ_H_, mult. (*J* in Hz)
1	137.6, C			
2	106.9, CH	6.59, d (1.5)	70.2, CH_2_	4.34, dd (11.2, 4.6)
				4.15, dd (11.2, 7.9)
3	157.1, C		48.2, CH	2.96, ddt (10.0, 7.9, 4.6)
4	112.9, C		200.8, C	
4a			104.4, C	
5	160.2, C		143.5, C	
6	101.3, CH	6.61, d (1.5)	132.6, C	
7	127.3, CH	6.85, d (16.2)	145.6, C	
8	128.5, CH	6.98, d (16.2)	131.4, C	
8a			142.3, C	
9			32.3, CH_2_	3.14, dd (13.9, 4.6)
				2.72, dd (13.9, 10.0)
4-CH_3_	8.5, CH_3_	2.04, s		
5-OCH_3_	56.0, CH_3_	3.84, s		
5-OH				11.40, s
6-OH				7.38, bs
7-OCH_3_			60.8, CH_3_	3.99, s
1′	130.6, C		129.7, C	
2′/6′	128.7, CH	7.38–7.34, m	131.0, CH	7.15–7.12, m
3′/5′	116.5, CH	6.79–6.75, m	116.2, CH	6.83–6.80, m
4′	158.3, C		157.0, C	
Ar–OH				8.24
Ar–OH				7.38

a In CD_3_OD.

b In CD_3_COCD_3_.

Compound **8** was isolated as a yellowish oil. A protonated molecule was found at *m*/*z* 333.0979 [M + H]^+^ (calcd. for C_17_H_17_O_7_^+^, 333.0969) in the HRESIMS. The aromatic part of the ^1^H NMR spectrum of **8** exhibited three signals for four protons on heteroatoms (δ_H_ 11.40, s; 8.24, bs; 7.38, bs) together with two characteristic signals of an AA′BB′ system at δ_H_ 7.15–7.12 and 6.83–6.80. The aliphatic part of the ^1^H NMR spectrum contained one methoxy group at δ_H_ 3.99 and five other signals whose spin–spin splitting pattern indicated that they belong to a single spin system (Table 1). Cross-peaks found in the HMBC spectrum suggested a structure similar to muscomin, which has a fully substituted ring A.^9^ However, the difference was the presence of one methoxy group instead of two methoxy groups. When the position of the methoxy group was determined, substitution at C-5 was excluded since a proton chemical shift of 11.40 ppm represented an intramolecular H-bond to the ketone (C-4). Moreover, this hydroxyl was correlated to C-4a, C-5, and C-6 in the HMBC spectrum (Figure 1). Also, a methyl substitution at 6-OH was ruled out by the ROESY correlation of 5-OH and 6-OH (δ_H_ = 7.38). However, the exact location of the methoxyl group at either C-7 or C-8 on the ring could not be deduced from the HMBC spectrum due to the substitutions on all positions. UV spectra were recorded in the presence of reagents since it was reported that boric acid in the presence of NaOAc forms a chelate with the *ortho*-dihydroxy groups at any locations on the flavonoid nucleus except for C-5 and C-6 and causes a 10–15 nm bathochromic shift in band II.^10,11^ Adding NaOAc and boric acid to the solution did not cause any bathochromic shift, suggesting the substitution of C-7.

For even greater certainty in determining the correct structure of **8**, the calculation of the theoretical NMR spectra for both positional isomers was employed. A conformational search was performed on both isomers of compound **8** using CREST and Grimme’s gfn2 method.^12,13^ Conformers were further optimized at the SMD(acetone)-B3LYP/6-31+G(d,p)^14−20^ level of theory to obtain the optimized geometry and energies of each conformer. In total, 48 conformers were identified for isomer A (C7-OCH_3_), while 38 conformers were identified for isomer B (C8-OCH_3_). Theoretical NMR calculations were conducted at the mPW1PW91/6-311+G(2d,p) scrf=(smd,solvent=acetone) level of theory.^14−21^ Scaling factors from the CHESHIRE Chemical Shift Repository^21−24^ were used in combination with a Boltzmann distribution to obtain the weighted chemical shifts to compare with experimental values (Table 2). The comparison indicated that compound **8** was likely the C7-methoxy derivative. NMR calculations were also performed in chloroform, the results of which are available in the Supporting Information (SI; Tables S2 and S3). While the results of NMR computations never provide 100% certainy of a product’s identity, the results reported here fall within previously accepted values for structure assignment.^21^

**Table 2:** Comparison of Experimental and Computed NMR Chemical Shift for **8**, **8a** (7-OCH_3_ Isomer), **8b** (8-OCH_3_ Isomer)^a^

	^13^C NMR			^1^H NMR		
no.	**8**	**8a**	**8b**	**8**	**8a**	**8b**
2	70.2	70.0	69.4	4.32	4.22	4.16
				4.14	4.14	4.04
3	48.2	50.9	49.8	2.95	2.86	2.91
4	200.8	199.1	198.3			
4a	104.4	102.5	100.8			
5	143.5	141.6	143.8			
6	**132.6**	130.3	**124.2**			
7	**145.6**	**141.8**	147.1			
8	131.4	129.7	127.0			
8a	142.3	139.8	149.5			
9	32.3	33.2	32.3	3.13	3.26	3.30
				2.71	2.63	2.55
7-OCH_3_	60.8	58.9	58.8	**3.98**	**4.12**	**3.67**
1′	129.7	131.4	131.4			
2′/6′	131.0	130.7	130.7	7.12	7.30	7.31
3′/5′	116.2	113.4	113.4	6.80	6.89	6.90
4′	157.0	154.8	154.8			
CMAD^b^		1.9	2.6		0.10	0.15
largest outliner^c^		Δδ 3.8	Δδ 8.4		Δδ 0.14	Δδ 0.31

a Hydroxyls were not compared due to the influence of several outside factors not considered during the computational study. For ^1^H multiplets, the median of the range is listed here.

b CMAD = corrected mean absolute deviation, computed as (1/*n*)∑*_i_^n^*|δ_comp_–δ_exp_| where δ_comp_ refers to the scaled computed chemical shifts.

c Largest outliers for each data set are highlighted in bold.

**Figure 2:**
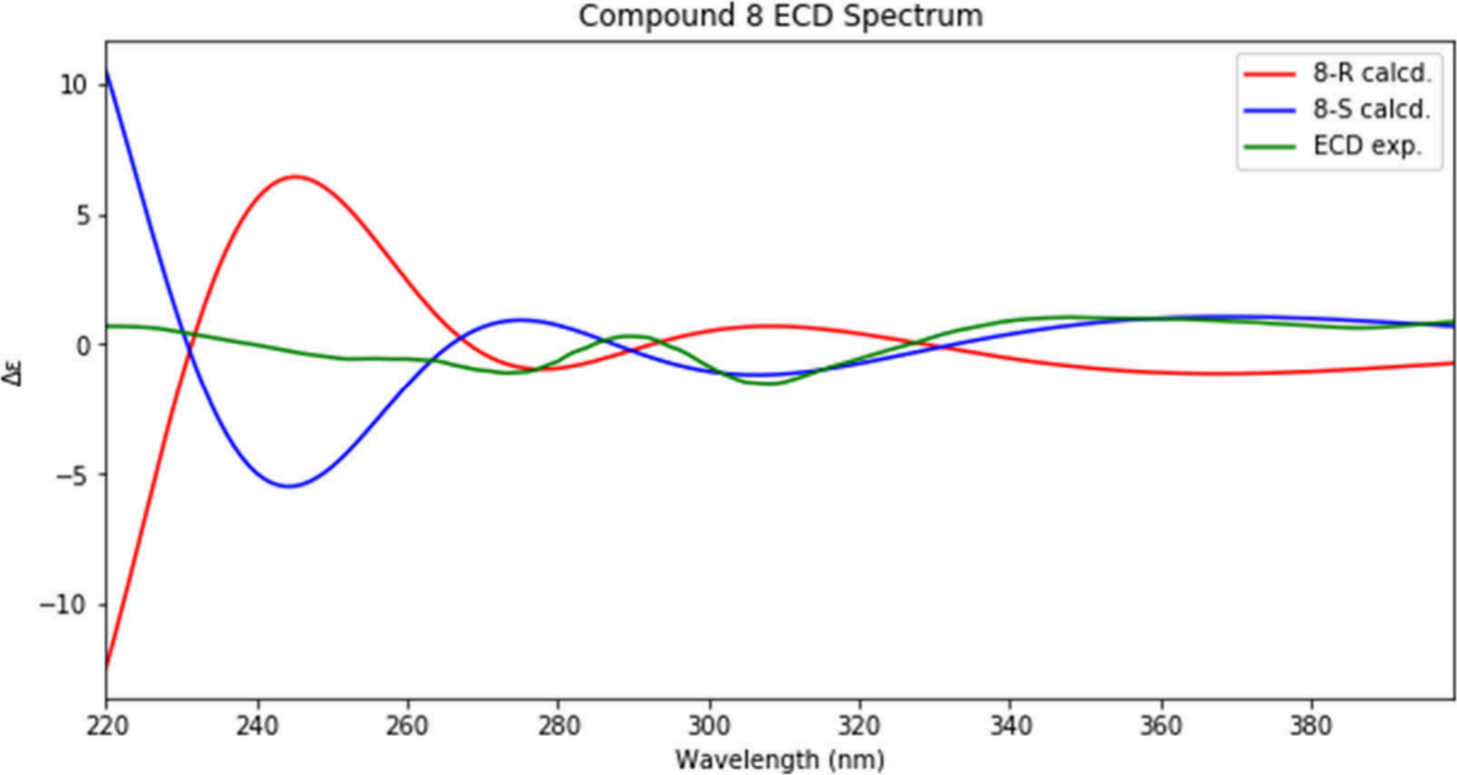
Experimental ECD curve of **8** (green) and calculated spectra of possible *R* (red) and *S* (blue) enantiomers.

The absolute stereochemistry of **8** was established using experimental and theoretical ECD analysis. ECD calculations were performed using TD-DFT with the SMD(methanol)-CAM-B3LYP/6-311+G(2d,p) level of theory.^25,26^ The *S* enantiomer had 48 conformers, while the *R* conformer had 51 (this difference is reflective of the lack of perfect reproducibility of the conformational searching procedure used). A graph comparing the experimental ECD results to thoe of the two enantiomers is shown in Figure 2. Based on all of the comparisons between the experimental and computed spectra, the structure of **8** was assigned as (3*S*)-(4′-hydroxybenzyl)-5,6,8-trihydroxy-7-methoxychroman-4-one and given the trivial name prosperin A.

**Figure 3:**
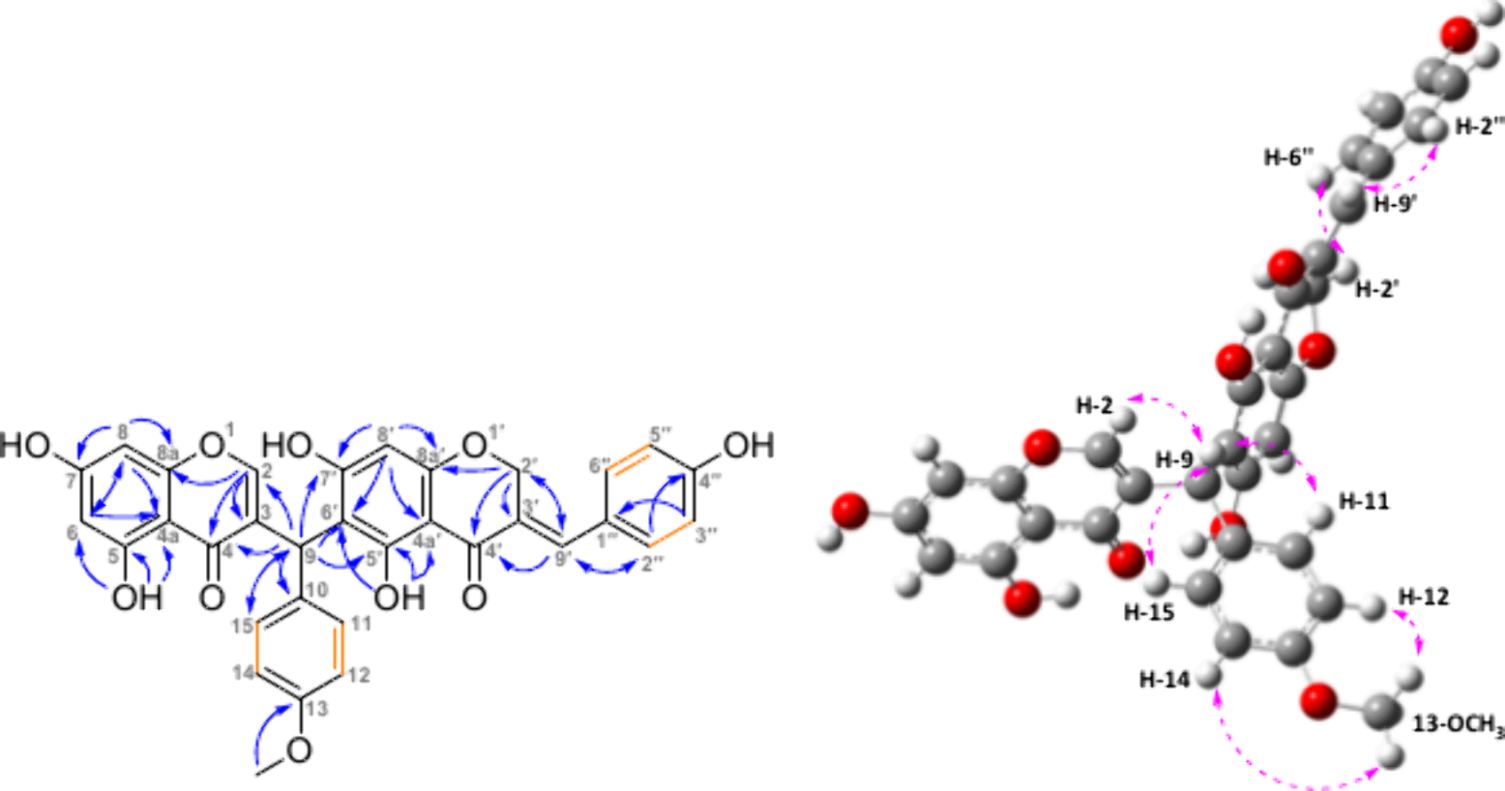
Left: Key HMBC correlations (blue arrows) and COSY correlations (orange bonds) of compound **9**. Right: Key NOESY correlations (pink dashed arrows) of compound **9**.

Compound **9** was obtained as a yellow, amorphous powder. The HRESIMS showed a molecular ion peak at *m*/*z* 603.1263 [M + Na]^+^ (calcd. for C_33_H_24_O_10_Na^+^, 603.1261), corresponding to the formula C_33_H_24_O_10_, when taken together with ^1^H and ^13^C NMR data (Table 3). The ^1^H NMR spectrum of **9** exhibited two deshielded sharp singlets characteristic for H-bonded protons (δ_H_ 13.63 and 12.78), one deshielded doublet with *J* = 1.1 Hz (δ_H_ 7.80), a multiplet at 7.75–7.73 ppm, two pairs of signals characteristic of AA′BB′ systems (δ_H_ 7.36–7.33, 7.24–7.21, 6.99–6.95, and 6.86–6.83), two *meta*-coupled aromatic protons with *J* = 2.1 Hz (δ_H_ 6.38 and 6.25), two singlets of methine groups (δ_H_ 5.99 and 5.97), a doublet of an oxymethylene group (δ_H_ 5.38, d, *J* = 1.8 Hz), and one characteristic singlet for an Ar-OCH_3_ group (δ_H_ 3.76). The ^13^C NMR spectrum contained 29 signals. Employing COSY, H2BC (Heteronuclear 2-Bond Correlation), HSQC, and HMBC experiments, two homoisoflavonoid fragments were identified, namely, (*E*)-4′-*O*-demethyleucomin^27^ and 5,7-dihydroxy-3-(4′-methoxybenzyl)chromone.^28^ All of the key correlations are depicted in Figure 3. The connection between the fragments was unambiguously identified to be C-9—C-6′ by the long-range couplings of H-9 to C-5′ (δ_C_ 164.2), C-6′ (δ_C_ 109.6), and C-7′ (δ_C_ 165.9). Unfortunately, elucidation of the stereochemistry has not been successful, even though there is only one chiral center. The compound was not isolated in sufficient purity to obtain a satisfactory comparison between experimental and computed ECD spectra (Figure S41). In addition, computed NMR shifts are provided in the SI (Table S4). Moreover, crystallization did not form crystals suitable for the X-ray analysis. It is possible that rotational isomers complicate this situation. To the best of our knowledge, compound **9**, named prosperin B, represents the first example of a dimeric homoisoflavonoid obtained from a natural source.

**Figure 4:**
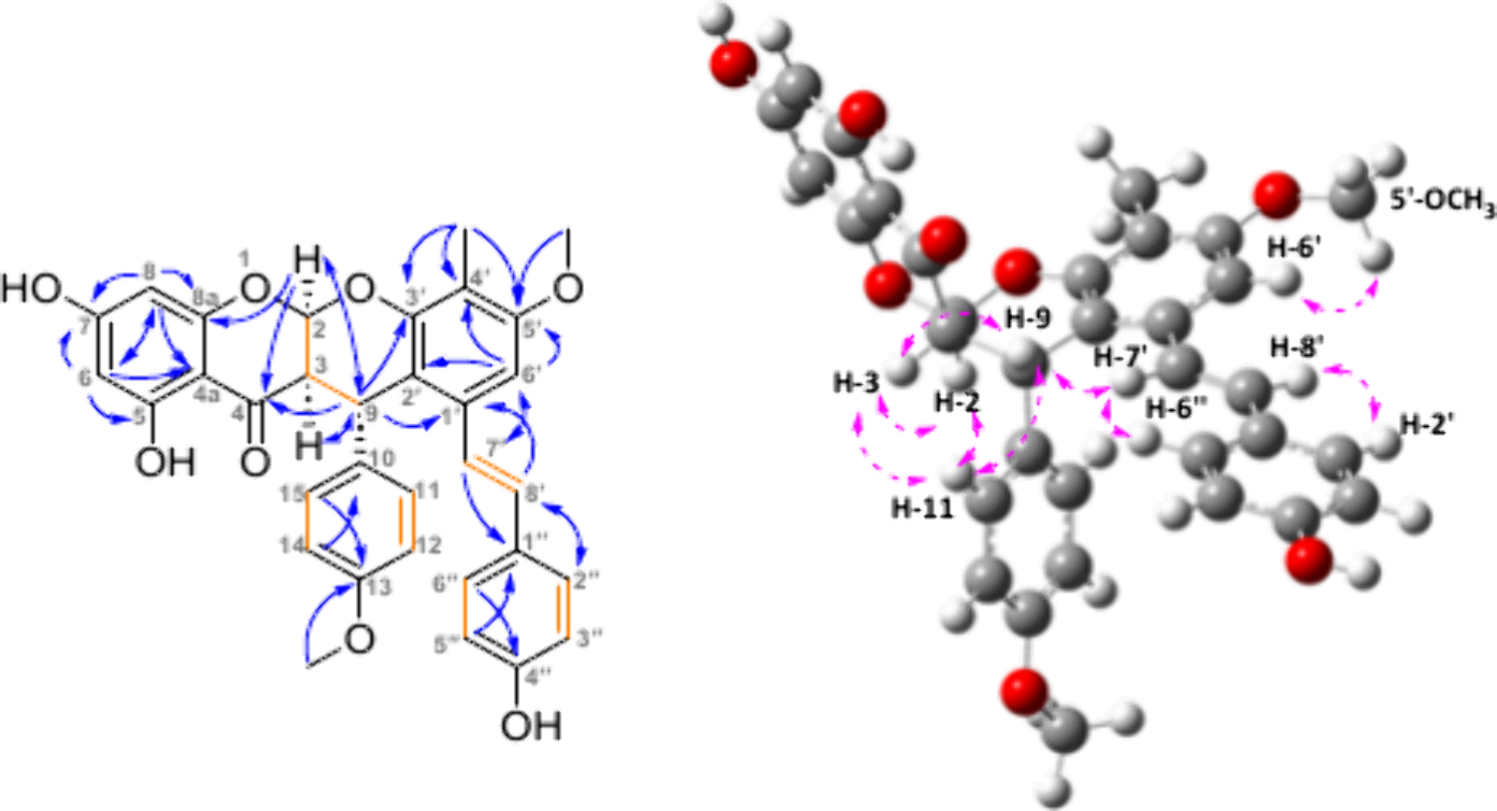
Left: Key HMBC correlations (blue arrows) and COSY and H2BC correlations (orange bonds) of compound **10**. Right: Key NOESY correlations (pink dashed arrows) are shown on an energy-minimized molecule **10** in Chem3D Pro (64-bit).

**Figure 5:**
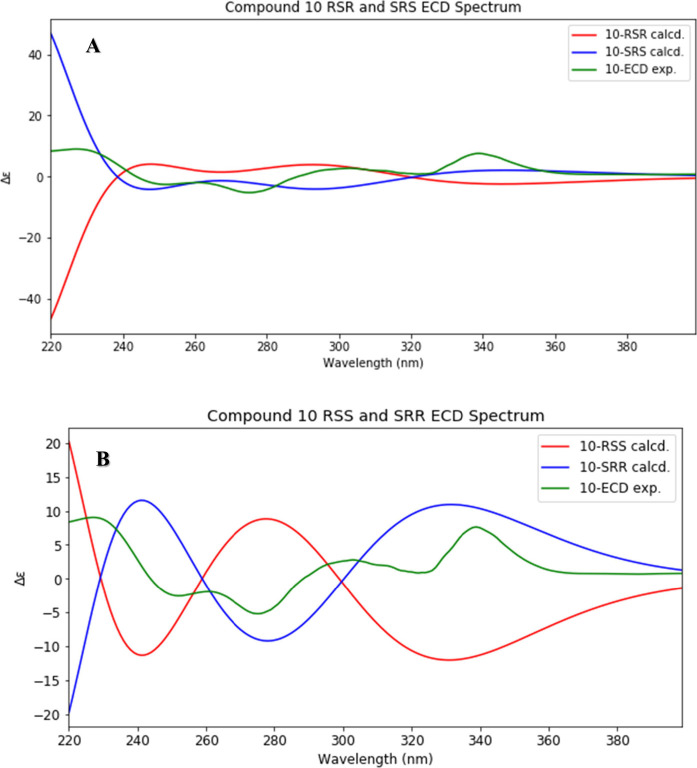
ECD spectra of compound **10** with experimental results (green), (A) *SRS* isomer (blue) and *RSR* isomer (red), and (B) *SRR* isomer (blue) and *RSS* isomer (red).

Compound **10** was obtained as a yellow, amorphous powder. Its molecular formula was established as C_33_H_28_O_8_ from HRESIMS, which exhibited a protonated molecular ion at *m*/*z* 553.1855 [M + H]^+^ (calcd for C_33_H_29_O_8_^+^, 553.1857), indicating 20 units of unsaturation. Regarding the signals in the ^1^H NMR spectrum (Table 3) of **10**, as with the previous compounds **8** and **9**, a signal characteristic of a H-bonded proton was observed (δ_H_ 11.79, s). Two pairs of characteristic signals for AA′BB′ systems, two doublets with a spin–spin interaction of *J* = 16.1 Hz representing a *trans* double bond (δ_H_ 7.03 and 6.96), a singlet at δ_H_ 6.98 overlapping with the mentioned doublet at δ_H_ 6.96, another two *meta*-coupled doublets with *J* = 2.1 Hz (δ_H_ 6.06 and 5.97), three signals arising from one spin–spin system (δ_H_ 5.94, d, *J* = 3.6 Hz, H-2; 3.64, dd, *J* = 3.6 and 2.3 Hz, H-3; 5.32, d, *J* = 2.3 Hz, H-9), two deshielded methoxy groups (δ_H_ 3.87 and 3.76), and one methyl group (δ_H_ 1.97) were present. The COSY and H2BC spectra confirmed the presence of the H-2/H-3/H-9 spin system. Employing the HMBC experiment, two fragments were identified, namely, homoisoflavonoid, 5,7-dihydroxy-3-(4′-methoxybenzyl)chromanone, and the new stilbenoid prospestilbene (**1**). The attachment points between homoisoflavonoid and stilbenoid were established following several lines of evidence. Deshielded ^1^H and ^13^C NMR chemical shifts of the sp^3^-methine group at position 2 pointed to the existence of an acetal, forming a C-2–O–C-3′ connection over an oxygen bridge. Furthermore, the key long-range correlations of H-9 (δ_H_ 5.32) with C-1′ (δ_C_ 135.9), C-4 (δ_C_ 193.2), C-3′ (δ_C_ 153.0), and also with C-6′ (δ_C_ 102.0) by a weak correlation over five bonds in a “W pattern,” revealed one of the attachment sites as C-9–C-2′. Cross-peaks found in the NOESY spectrum confirmed the suggested constitution of molecule **10** (see Figure 4). Moreover, the NOESY experiment and careful analysis of spin–spin couplings provided a relative configuration. Namely, the splitting pattern with small *J*-couplings and NOESY cross-peaks of H-2/H-3 indicated that they point to the same side of this tetracycle. The configuration at C-9 was determined by the NOESY correlations of H-11/H-15 belonging to the *para*-disubstituted system. These protons on sp^2^ hydridized carbons also showed cross-peaks with H-2 and H-3, which require the benzene ring to point to the same side of the tetracycle as those CH groups. Also, H-9 showed a correlation with H-7′. All of the NOESY correlations are shown in Figure 4. Taken together, these findings revealed the relative configuration. To elucidate the absolute configuration, calculations for the ECD spectra were performed with the same method as that described for compound **8**. Two configurations showed a comparable match, (2*S*,3*R*,9*S*)-**10**, and (2*S*,3*R*,9*R*)-**10**. Both of these configurations, their enantiomers, and experimental ECD results are shown in Figure 5. Other enantiomer pairs can be seen in the SI (Figure S42).

**Table 3:** ^1^H (500 MHz) and ^13^C (125.7 MHz) NMR Data of **9** and **10** in CD_3_COCD_3_

	**9**		**10**	
no.	δ_C_, type	δ_H_, mult. (*J* in Hz)	δ_C_, type	δ_H_, mult. (*J* in Hz)
2	157.3, CH	7.78, d (1.1)	95.7, CH	5.94, d (3.6)
3	125.1, C		50.0, CH	3.64, dd (3.6, 2.3)
4	182.2, C		193.2, C	
4a	105.6, C		102.3, C	
5	163.5, C		164.9, C	
6	99.8, CH	6.25, d (2.1)	97.8, CH	5.97, d (2.1)
7	165.1, C		168.6, C	
8	94.4, CH	6.38, d (2.1)	97.0, CH	6.06, d (2.1)
8a	159.12, C		160.1, C	
9	35.9, CH	5.97, bs	36.9, CH	5.32, d (2.3)
10	133.2, C		135.8, C	
11/15	130.2, CH	7.24–7.21, m	130.1, CH	7.20–7.16, m
12/14	114.3, CH	6.86–6.83, m	115.1, CH	6.96–6.92, m
13	159.08, C		159.5, C	
5-OH		12.76, s		11.79, s
7-OH		10.00–9.00, bs		b
13-OCH_3_	55.4, CH_3_	3.76, s	55.4, CH_3_	3.76, s
1′			135.9, C	
2′	68.1, CH_2_	5.38, d (1.8)	112.3, C	
3′	128.1, C		153.0, C	
4′	185.9, C		113.0, C	
4a′	103.2, C			
5′	164.2, C		158.3, C	
6′	109.6, C		102.0, CH	6.98, s^a^
7′	165.9, C		123.2, CH	6.96, d (16.1)^a^
8′	96.1, CH	5.99, s	130.8, CH	7.03 d (16.1)
8a′	161.8, C			
9′	137.5, C	7.75–7.73, m		
4′-CH_3_			8.6, CH_3_	1.97, s
5′-OH		13.62, s		
5′-OCH_3_			55.9, CH_3_	3.87, s
7′-OH		10.00–9.00, bs		
1″	126.7, C		130.0, C	
2″/6′′	133.5, CH	7.36–7.33, m	128.7, CH	7.26–7.22, m
3″/5′′	116.7, CH	6.99–6.95, m	116.3, CH	6.80–6.76, m
4″	160.1, C		158.2, C	
4″-OH		b		b

a Overlapped.

b Not observed.

To assign the absolute configuration for compound **10**, computational NMR was conducted for isomers *SRS* and *SRR* following the same methods as those used for compound **8**. A table showing the experimental chemical shifts and calculated shifts for ^1^H and ^13^C NMR spectra is shown in Figure S43. Overall, 2*S*,3*R*,9*S* is a better assignment in large part due to the assignment for H-9, which has a much worse match compared to the same hydrogen for 2*S*,3*R*,9*R* (difference of ∼0.1 ppm in 2*S*,3*R*,9*S*, while it is >0.5 ppm off in 2*S*,3*R*,9*R*). Such a significant difference is more than enough to have a high degree of confidence that 2*S*,3*R*,9*S* is the better assignment of the two. Combined with the ECD data and the experimental *J*-coupling of this compound, we have fairly good confidence in our assignment for this product being 2*S*,3*R*,9*S*-**10**. The novel homoisoflavone-stilbene heterodimer **10** was thus named prosperin C.

The other six known compounds were identified as pinostilbene (**2**),^29^ scillabene A (**3**),^30^ (*E*)-4′-*O*-demethyleucomin (**4**),^27^ 4′-demethyl-3,9-dihydroeucomin (**5**),^31^ 3,9-dihydropunctatin (**6**),^32^ and 3*R*-(4′-hydroxybenzyl)-6,8-dihydroxy-5,7-dimethoxy-4-chromanone (**7**)^33^ by comparing their spectroscopic data with those reported in the literature. All compounds are reported here from *P. autumnale* for the first time.

All isolated compounds were tested for their potential cytotoxic activities against eight different human cancer cell lines including colon (HCT116 and LoVo), prostate (DU145 and PC3), liver (HEP3B and HEPG2), and breast (MCF7 and MDA-MB-231) as well as against a nontumor mouse fibroblast cell line (L929) by MTS assay (Table 4). Doxorubicin was used as a positive control. Among the tested compounds, **1**, **2**, **4**, and **10** exhibited potent cytotoxicity toward one or more cell lines with the IC_50_ values ranging from 8.2 to 37.6 μM, while **5**–**8** did not cause inhibition (IC_50_ > 100 μM) of the proliferation of cancer cells at the tested concentration. Interestingly, for the stilbenoid structures **1**–**3**, there was a significant difference in growth suppression, with compound **3** being the least effective. This may be due to increased polarity by the trihydroxy substitution, which may prevent the compound from entering the cells. This is the first study reporting any biological effect of **3**. The only previous study focused on the isolation and characterization of the molecule.^30^

**Table 4:** Cytotoxic Activities of Compounds **1**–**10**^c^ (IC_50_ μM)

	IC_50_ ± SEM^a^								
compound	HCT116	LoVo	MCF7	MDA-MB-231	PC3	DU145	HEP3B	HEPG2	L929
**1**	54.6 ± 5.5	10.6 ± 0.5	53.5 ± 9.1	79.6 ± 5.6	21.3 ± 2.1	29.7 ± 4.4	59.8 ± 4.2	37.6 ± 5.3	33.7 ± 3.9
**2**	71.3 ± 12.6	8.2 ± 0.4	95.7 ± 15.0	23.3 ± 1.4	88.7 ± 10.9	>100	93.5 ± 8.9	51.8 ± 10.0	13.4 ± 1.7
**3**	>100	83.4 ± 3.9	>100	>100	>100	>100	82.7 ± 3.9	>100	>100
**4**	45.8 ± 8.4	40.4 ± 2.9	95.3 ± 4.7	77.5 ± 3.9	88.1 ± 8.6	>100	33.7 ± 3.0	>100	>100
**9**	75.8 ± 8.0	>100	>100	>100	>100	>100	>100	>100	>100
**10**	19.3 ± 3.1	16.7 ± 0.8	25.2 ± 5.1	25.5 ± 2.4	36.7 ± 2.5	40.1 ± 2.8	59.9 ± 6.9	24.0 ± 2.2	29.7 ± 1.7
doxorubicin^b^	0.9 ± 0.1	2.4 ± 0.2	2.3 ± 0.3	2.3 ± 0.3	0.9 ± 0.1	5.9 ± 0.4	1.1 ± 0.1	4.5 ± 0.3	>100

a IC_50_ for compounds **5**–**8**: >100 μM. IC_50_ values were calculated from the cell growth inhibition curves obtained from the treatments with increasing concentrations of compounds for 48 h. Experiments were carried out in triplicate.

b Positive control.

c Compounds **8** and **9** have a purity of less than 95%. IC_50_ values of compounds selected for mechanistic studies are marked in **bold**.

The results indicated that especially LoVo, MDA-MB-231, PC-3, DU145, and HEPG2 were the most sensitive cancer cells to the tested compounds (Table 4). On the other hand, when the IC_50_ values in cancer and healthy (L929) cell lines were compared, it could be speculated that compound **1** seems to be more selective for killing LoVo (IC_50_ = 10.6 ± 0.5), PC-3 (IC_50_ = 21.3 ± 2.1), and DU145 (IC_50_ = 29.7 ± 4.4), while **2** was only selective for LoVo (IC_50_ = 8.2 ± 0.40). Likewise, compound **4** had a higher selectivity for HEP3B (IC_50_ = 33.7 ± 3.0), whereas **10** was slightly selective for HCT116 (19.3 ± 3.1), LoVo (16.7 ± 0.8), MCF7 (25.2 ± 5.1), MDA-MB-231 (25.5 ± 2.4), and HEPG2 (24.0 ± 2.2) cells.

**Figure 6:**
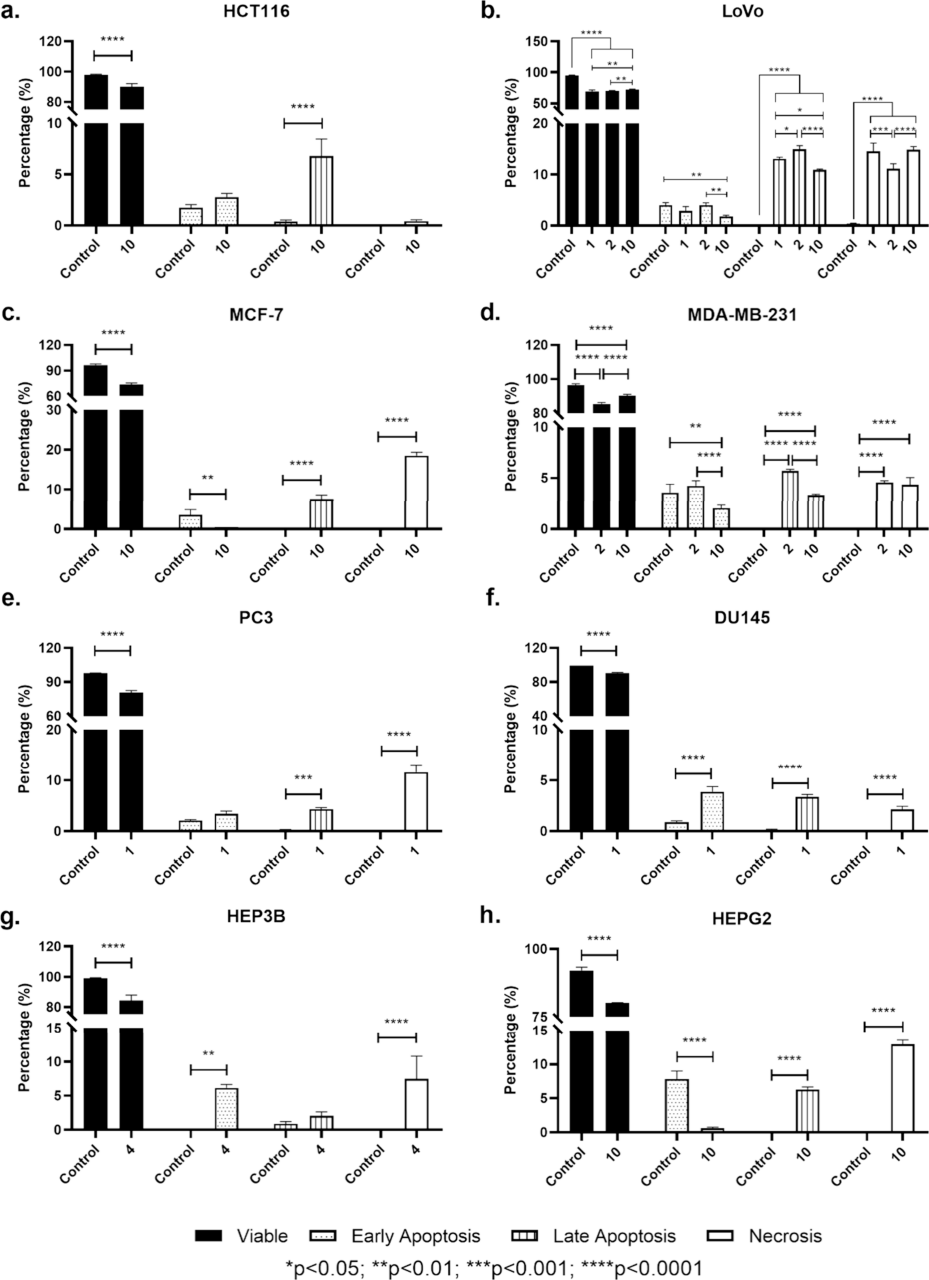
Bar graphs indicate the differences between the control and compounds **1**, **2**, **4**, and **10** in relevant cell lines tested in terms of viability, early apoptosis, late apoptosis, and necrosis.

**Figure 7:**
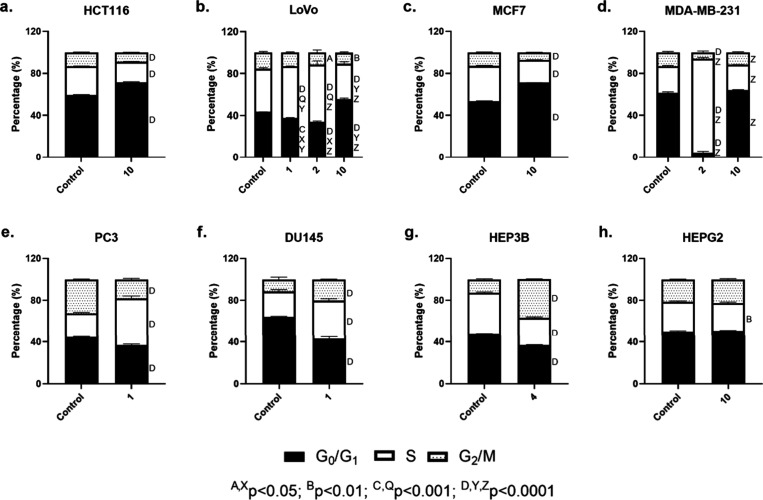
Bar graphics indicate the differences between the control and compounds **1**, **2**, **4**, and **10** in related cell lines tested in terms of the phases of the cell cycle.

Based on the cytotoxicity results, we decided to deeply investigate the cell death mechanisms of the most active ones (**1**, **2**, **4**, and **10**) in the relevant cancer cell lines at their IC_50_ values. Thus, these compounds were evaluated for their apoptotic and necrotic effects as well as their arrest potential on cell cycle progression in related cancer cell lines. The results are presented in Figures 6 and 7. All tested compounds significantly reduced viability in all cell lines tested (*p* < 0.05). In terms of colorectal cancer cell lines (HCT116 and LoVo), the decrease in viability was accompanied by a significant increase in apoptosis in HCT116 cells for **10** and apoptosis along with necrosis in LoVo cells (*p* < 0.05) for **1**, **2**, and **10**. In breast cancer cell lines (MCF7 and MDA-MB-231), **10** increased apoptosis and necrosis in both cells, though in MDA-MB-231 cells, **2** was more efficient in inducing apoptosis compared to **10** (*p* < 0.05). For both prostate cancer cell lines (PC3 and DU145), **1** exerted anticancer action via promoting apoptosis and necrosis compared to control cells (*p* < 0.05). In terms of hepatocellular carcinoma cell lines, **4** significantly increased early apoptosis along with necrosis in HEP3B cells (*p* < 0.05), while **10** led to higher late apoptosis and necrosis in HEPG2 cells.

Regarding cell cycle analyses (Figure 7), **1** led to S phase arrest in the LoVo (*p* < 0.05), PC3 (*p* < 0.05), and DU145 (*p* < 0.05) cell lines, with G2/M phase arrest accompanying the latter (*p* < 0.05). Compound **2** induced S phase arrest in LoVo and MDA-MB-231 cell lines, along with decreasing G2/M phases. Compound **4** promoted the G2/M phase arrest in HEP3B cells and decreased G0/G1 as well as S phases, while **10** led to G0/G1 phase arrest in HCT116, LoVo, MCF7, and MDA-MB-231 cell lines, although it resulted in a slight S phase decrease in the HEPG2 cell line (*p* < 0.01).

## Conclusions

In conclusion, 10 phenolic compounds, including four previously undescribed compounds (prospestilbene and prosperins A–C) were obtained from the bulbs of *P. autumnale* through cytotoxic activity-guided fractionation. The structures of the isolated compounds were elucidated using NMR, IR, and UV spectroscopy, as well as HRESIMS and chiroptical methods. This is the first report on the isolation of a homoisoflavone-stilbene heterodimer and a dimeric homoisoflavonoid from a natural source. Among the purified metabolites, prospestilbene (**1**), pinostilbene (**2**), (*E*)-4′-*O*-demethyleucomin (**4**), and prosperin C (**10**) seem to be the secondary metabolites that are mainly responsible for the *in vitro* cytotoxic effect of *P. autumnale* extracts. They inhibited the proliferation of tested cancer cell lines (IC_50_ 8.2–37.6 μM) through inducing apoptosis, necrosis, or cell cycle arrest. Therefore, **1**, **2**, **4**, and **10** deserve further attention as well as *in vivo* testing, as they could be potential anticancer leads in drug discovery.

## Experimental Section

### General Experimental Procedures

Optical rotation was measured on a KRÜSS optronic P3000 automatic polarimeter in MeOH. The ECD spectra were recorded on a JASCO J-815 CD spectrometer in MeOH. UV spectra were recorded using an HP Agilent 8453 UV–vis spectrophotometer (Agilent Technologies, Santa Clara, CA, USA). IR spectra (υ in cm^–1^) were recorded on a Nicolet iS50 FTIR (Thermo Scientific). NMR spectra were recorded on VNMR S500 (Varian) spectrometers at 500 MHz for proton nuclei and 125.7 MHz for carbon nuclei at 25 °C. CD_3_OD was referenced to 3.30 ppm for ^1^H NMR data and 49.0 ppm for ^13^C NMR data, and CD_3_COCD_3_ was referenced to 2.06 ppm for ^1^H NMR data and 29.8 ppm for ^13^C NMR data. The HRESIMS data were obtained using an Acquity UPLC-I Class UHPLC system (Waters, Milford, USA) coupled with a Synapt G2-Si high-resolution mass spectrometer (Waters, Manchester, UK) based on a Q-TOF platform.

For column chromatography (CC), silica gel 60 (0.063–0.200 mm; Merck, Darmstadt, Germany) and Sephadex LH-20 (25–100 μm; Sigma-Aldrich, St. Louis, MO, USA) were utilized. Medium-Pressure Liquid Chromatography (MPLC) was carried out using a Sepacore Flash Systems X10/X50 (Buchi Labortechnik AG, Flawil, Switzerland) on RediSep Gold columns LiChroprep C_18_, 5.5, 15.5, and 150 g; SiO_2_, 12 and 120 g (Teledyne Isco, Lincoln, Nebraska, USA). TLC analyses were carried out on precoated silica gel 60 F_254_ plates (9.5–11.5 μm; Merck, Darmstadt, Germany). TLC plates were derivatized by spraying vanillin/H_2_SO_4_ solution (1%) followed by heating at 105 °C for 2–3 min, then visualized under UV light at 254 and 366 nm.

### Plant Material

The bulbs of *Prospero autumnale* (Asparagaceae) were collected from Kayışdağı, İstanbul (40° 58′ 16.876″ N, 29° 8′ 54.324″ E), in September 2022. The plant material was identified by Prof. Dr. Hasan Kırmızıbekmez. The voucher specimen (YEF 22044) was deposited at the Herbarium of the Faculty of Pharmacy, Yeditepe University, İstanbul, Türkiye.

### Extraction and Isolation

The air-dried and minced bulbs of *P. autumnale* (210 g) were macerated with absolute EtOH (≥99.9%, 2 L) for 2 days and then extracted two times at 45 °C to yield a crude EtOH extract (12.2 g, yield: 5.8%) after the solvent removal on a rotavapor. The crude EtOH extract was suspended in 85 mL of MeOH (10%) and then partitioned successively with equal volumes (each, 85 mL × 3) of EtOAc and *n*-BuOH, respectively, giving EtOAc (6.19 g), *n*-BuOH (2.19 g), and H_2_O (3.70 g) fractions. The EtOAc fraction (6.1 g), which displayed significant cytotoxic activity (Table S1), was selected to isolate potential cytotoxic compounds from cancer cell lines. This fraction was separated through a SiO_2_ CC (70 g) eluting with *n*-hexane-EtOAc (0–100%, in steps of 20%) and EtOAc-MeOH (50:50) mixtures to obtain six main fractions (frs. A–F). Fr. B (450 mg) was applied to SiO_2_ CC (70 g) using an *n*-hexane-EtOAc mixture (90:10 → 50:50) as a mobile phase to obtain four main fractions, frs. B_1–4_. Fr. B_2_ (160 mg) was loaded to a Sephadex LH-20 CC (22 g) eluting with CH_2_Cl_2_-MeOH (50:50) to obtain fr. B_2B_ (86 mg), which was further purified by C_18_-MPLC (15.5 g) with a gradient of MeOH (30–50%) to give **1** (3 mg). Purification of fr. B_4_ (25 mg) with a Sephadex LH-20 CC (22 g) eluting with CH_2_Cl_2_-MeOH (50:50) yielded **4** (6 mg) and **5** (3 mg). Fr. C (2.1 g) was applied to SiO_2_-MPLC (120 g) with a gradient of *n*-hexane-EtOAc (85:15 → 50:50) to give 10 subfractions, frs. C_1_–_10_. Fr. C_1_ (27 mg) was subjected to Sephadex LH-20 CC (10 g) eluting with MeOH to afford **2** (6 mg) and **5** (6 mg). Similarly, compounds **4** (13 mg) and **6** (9 mg) were purified from fr. C_3_ (65 mg) using a Sephadex LH-20 CC (35 g) eluting with MeOH. Compound **3** (13 mg) was isolated from fr. C_4_ (48 mg) using a Sephadex LH-20 CC (35 g) and MeOH. Similarly, fr. C_7_ (130 mg) was fractionated with Sephadex LH-20 CC (35 g), eluting with MeOH to give three fractions, frs. C_7A-C_. Among these fractions, fr. C_7B_ (20 mg) was applied to C_18_-MPLC (5.5 g) with a gradient of MeOH (40 → 90%) to yield **10** (7 mg). Fr. C_9_ (397 mg) was loaded onto Sephadex LH-20 CC (55 g) and eluted with MeOH to give fr. C_9B_ (14 mg), which was further purified by SiO_2_-MPLC (12 g) with a gradient of *n*-hexane-EtOAc (80:20 → 45:55) to purify **9** (5 mg). Fr. C_10_ (166 mg) was likewise separated by Sephadex LH-20 CC (35 g), eluting with MeOH to purify **8** (8 mg). Fr. D (1.19 g) was subjected to C_18_-MPLC (150 g) with a gradient of MeOH in H_2_O (10 → 80% MeOH) to obtain six main subfractions, frs. D_1–6_. Among them, fr. D_4_ (70 mg) was applied to Sephadex LH-20 CC (55 g), eluting with MeOH to give fr. D_4A_ (35 mg), which was further repeatedly purified by SiO_2_-MPLC (12 g) with a gradient of CHCl_3_-MeOH (100:0 → 90:10) and *n*-hexane-EtOAc (70:30 → 20:80) to yield **7** (15 mg).

Prospestilbene (**1**): Pale amorphous powder. UV (MeOH): λ_max_ 212, 239 (sh), 311, 324 nm. IR (KBr): υ_max_ 3187, 1603, 1584, 1513 cm^–1^. For ^1^H and ^13^C NMR data, see Table 1. HRESIMS, *m*/*z* calcd. for C_16_H_17_O_3_^+^ [M + H]^+^: 257.1172. Found: 257.1177. See the SI for UV, IR, NMR, and HRMS spectra in Figures S1–S8.

Prosperin A (**8**): Yellowish oil. [*a*]_D_^29^ = +112.7 (*c* 0.10, MeOH). ECD (0.2 mg/mL, MeOH): Δε_273_ = −0.55, Δε_308_ = −0.76. UV (MeOH): λ_max_ 223, 278 nm. IR (KBr): υ_max_ 3216, 1706, 1614, 1515 cm^–1^. For ^1^H and ^13^C NMR data, see Table 1. HRESIMS, *m*/*z* calcd. for C_17_H_17_O_7_^+^ [M + H]^+^: 333.0969. Found: 333.0979. See the SI for ECD, UV, IR, NMR, and HRMS spectra in Figures S9–S19.

Prosperin B (**9**): Yellow amorphous powder. [*a*]_D_^23^ = +9.2 (*c* 0.26, MeOH). ECD (0.2 mg/mL, MeOH): Δε_237_ = +0.69, Δε_260_ = +1.20, Δε_287_ = +0.66, Δε_317_ = +2.38, Δε_343_ = +5.20. UV (MeOH): λ_max_ 230 (sh), 258 (sh), 296 (sh), 364 nm. IR (KBr): υ_max_ 3364, 1628, 1582, 1510 cm^–1^. For ^1^H and ^13^C NMR data, see Table 3. HRESIMS, *m*/*z* calcd. for C_33_H_24_O_10_Na^+^ [M + Na]^+^: 603.1261. Found: 603.1263. See the SI for ECD, UV, IR, NMR, and HRMS spectra in Figures S20–S29.

Prosperin C (**10**): Yellow amorphous powder. [*a*]_D_^23^ = +185.0 (*c* 0.17, MeOH). ECD (0.2 mg/mL, MeOH): Δε_227_ = +7.56, Δε_252_ = −2.13, Δε_275_ = −4.34, Δε_339_ = +6.38. UV (MeOH): λ_max_ 211, 297, 322 nm. IR (KBr): υ_max_ 3346, 1637, 1603, 1510, 1463 cm^–1^. For ^1^H and ^13^C NMR data, see Table 3. HRESIMS, *m*/*z* calcd. for C_33_H_29_O_8_^+^: [M + H]^+^ 553.1857. Found: 553.1855. See the SI for ECD, UV, IR, NMR, and HRMS spectra in Figures S30–S40.

### Cytotoxicity Assay

To determine the *in vitro* cytotoxicity of the extracts and compounds, colorectal (HCT116 and LoVo), breast (MCF7 and MDA-MB-231), prostate (PC3 and DU145), and liver (HEP3B and HEPG2) cancer cell lines in addition to a nontumor mouse fibroblast cell line (L929) were seeded at 5 × 10^3^ cells/well in triplicate into 96-well tissue culture plates and incubated overnight in an incubator at 37 °C under a humid environment to allow attachment. Stock solutions of extracts and compounds were prepared by dissolving in DMSO (Sigma-Aldrich, no. D2650) at the concentrations of 100 mg/mL and 100 mM for extracts and compounds, respectively. Further dilutions were performed in the following complete culture media: DMEM/F12 medium (Thermo Fisher Scientific, #11320033) for colorectal cancer cell lines and DMEM medium (Capricorn Scientific, #DMEM-HA) for other cell lines used in this study, which are both supplemented with 10% Fetal Bovine Serum (FBS; Sigma-Aldrich, #F7524) and 1% penicillin-streptomycin antibiotic solution (Capricorn Scientific, #PS-B). The final DMSO content in the media did not exceed 0.1%, which is safe for cell culture studies. After attachment, cells were treated with the extracts at the concentrations of 3.12, 6.25, 12.5, 25, 50, and 100 μg/mL or isolated compounds at 3.12, 6.25, 12.5, 25, 50, and 100 μM for 48 h. Untreated cells were used as a control, while plain culture medium was used as a blank. Viability was determined with MTS Assay (Abcam #ab197010) according to the manufacturer’s instructions. Absorbance at 490 nm was measured with a spectrophotometer (Epoch, BioTek Instruments). Relative viability ratios were calculated with the equation given below, and IC_50_ values were determined in GraphPad Prism (version 8.0).
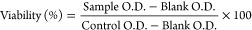


### Annexin V/Propidium Iodide Staining

Apoptosis, necrosis, and viability were analyzed by Annexin V-FITC and thiocyaniodide (PI) staining. For this purpose, cells were seeded at 5 × 10^5^ cells per 60 mm dish and incubated overnight to allow attachment. Then, cells were treated with the selected compounds (**1**, **2**, **4**, and **10**) for 48 h at respective IC_50_ concentrations. For flow cytometric analysis, cells were detached with Trypsin-EDTA solution (Thermo Fisher Scientific #25200056), washed with Dulbecco’s phosphate buffered saline (DPBS, Thermo Fisher Scientific #14190094) solution, and suspended in 1 mL of ice-cold Annexin V Binding Buffer (BioLegend #422201). Cells were then stained with 1 μL of Annexin V-FITC (Biovision #1001) and 1 μL PI (Thermo Fisher Scientific # P1304MP, diluted to 250 μg/mL in DPBS) by incubating cells on ice for 15 min in the dark. Cells were then immediately analyzed on a Beckman Coulter DxFLEX flow cytometry system. Each analysis was performed in triplicate, and 25 × 10^3^ events were counted for each tube. Evaluations were done with CytEXPERT software.

### DNA Content Analysis

To investigate cell cycle progression, cells were seeded as 5 × 10^5^ cells per 60 mm dish and incubated overnight for attachment. After incubating with the selected compounds for 48 h, cells were detached with Trypsin-EDTA, washed once with DPBS, and fixed with ice-cold 70% EtOH by storing tubes at 4 °C for an hour. Then, EtOH was discarded by centrifuging cells, and the pellet was suspended in 500 μL cell cycle kit reagent (Beckman Coulter #C03551). Tubes were incubated at room temperature under the dark for 30 min. The experiment was done on triplicate, and 5 × 10^4^ cells per test were acquired at a medium flow rate with the Beckman Coulter DxFLEX flow cytometry system. Analyses were performed with ModFit LT version 4.0.

## Data Availability

The NMR data for **1** and **8**–**10** have been deposited in the Natural Products Magnetic Resonance Database (NP-MRD; www.np-mrd.org) and can be found at NP0341798–NP0341801.

## Supporting Information Available

Spectroscopic and spectrometric data and quantum chemical calculations for new compounds (PDF)
