# Supplementary material for: Cytotoxic Stilbenoids, Hetero- and Homodimers of Homoisoflavonoids from Prospero autumnale
Source: J Nat Prod. 2025 Jan 24;88(2):458–68. doi: 10.1021/acs.jnatprod.4c01263 (PMC11877502; doi:10.1021/acs.jnatprod.4c01263)
Supplement: Supplementary file 1 — np4c01263_si_001.pdf [file np4c01263_si_001.pdf]

Supporting information for:

Cytotoxic Stilbenoids, Hetero- and Homodimers of Homoisoflavonoids from *Prospero autumnale*

Hasan Kırmızıbekmez,*,† Başak Aru,‡ Jana Křoustková,*,§ Murat Erdoğan,† Ian Torrence,ǁ Kaori Ando,ǁ Dean J. Tantillo,ǁ Milan Malaník,⊥ Štefan Kosturko,§,∇ Jiří Kuneš,# and Lucie Cahlíková§

†Department of Pharmacognosy, Faculty of Pharmacy, Yeditepe University, TR−34755, Kayışdağı, İstanbul, Türkiye

‡Department of Immunology, Faculty of Medicine, Yeditepe University, TR−34755, Kayışdağı, İstanbul, Türkiye

§Department of Pharmacognosy and Pharmaceutical Botany, Faculty of Pharmacy, Charles University , Heyrovského 1203, 500 03 Hradec Kralove, Czech Republic

ǁDepartment of Chemistry, University of California, Davis, California 95616, United States

⊥Department of Natural Drugs, Faculty of Pharmacy, Masaryk University, Palackého třída 1946/1, 61200 Brno, Czech Republic

#Department of Organic and Bioorganic Chemistry, Faculty of Pharmacy, Charles University, Heyrovského 1203, 500 03 Hradec Kralove, Czech Republic

∇Department of Analytical Chemistry, Faculty of Pharmacy, Charles University , Heyrovského 1203, 500 03 Hradec Kralove, Czech Republic

*E−mail: hasankbekmez@yahoo.com *E−mail: marikoj2@faf.cuni.cz

Table of Contents

Scheme S1.1 Isolation of compounds from *Prospero autumnale*. S4

Scheme S1.2 Isolation of compounds from *Prospero autumnale.* S5

Table S1. Cytotoxic activities of the extract and fractions[a] S6

Figure S1. HRESIMS of prospestilbene (1) S7

Figure S2. ^{1}H NMR (500 MHz) spectrum of prospestilbene (1) in CD_3OD S7

Figure S3. ^{13}C NMR (125.7 MHz) spectrum of prospestilbene (1) in CD_3OD S8

Figure S4. HSQC spectrum of prospestilbene (1) S8

Figure S5. HMBC spectrum of prospestilbene (1) S9

Figure S6. NOESY spectrum of prospestilbene (1)... S10

Figure S7. IR spectrum of prospestilbene (1) S10

Figure S8. UV spectrum of prospestilbene (1) S11

Figure S9. HRESIMS of prosperin A (8) S12

Figure S10. ^{1}H NMR (500 MHz) spectrum of prosperin A (8) in CD_3COCD_3................. S12

Figure S11. ^{13}C NMR (125.7 MHz) spectrum of prosperin A (8) in CD_3COCD_3 .... S13

Figure S12. HSQC spectrum of prosperin A (8).. S13

Figure S13. HMBC spectrum of prosperin A (8) S14

Figure S14. ROESY spectrum of prosperin A (8) S14

Figure S15. ^{1}H NMR (600 MHz) spectrum of prosperin A (8) in $CDCl_3$ S15

Figure S16. ^{13}C NMR (150 MHz) spectrum of prosperin A (8) in $CDCl_3$ S15

Figure S17. ECD spectrum of prosperin A (8) in MeOH S16

Figure S18. IR spectrum of prosperin A (8) S16

Figure S19. UV spectrum of prosperin A (8) S16

Figure S20. HRESIMS of prosperin B (9)... S17

Figure S21. ^{1}H NMR (500 MHz) spectrum of prosperin B (9) in CD_3COCD_3................. S17

Figure S22. ^{13}C NMR (125.7 MHz) spectrum of prosperin B (9) in CD_3COCD_3............. S18

Figure S23. HSQC spectrum of prosperin B (9).. S19

Figure S24. COSY spectrum of prosperin B (9).. S19

Figure S25. HMBC spectrum of prosperin B (9)... S20

Figure S26. NOESY spectrum of prosperin B (9) S20

Figure S27. ECD spectrum of prosperin B (9) in MeOH S21

Figure S28. IR spectrum of prosperin B (9) S21

Figure S29. UV spectrum of prosperin B (9)... S21

Figure S30. HRESIMS of prosperin C (10) S22
Figure S31. ^{1}H NMR (500 MHz) spectrum of prosperin C (10) in CD_3COCD_3 .... S22
Figure S32. ^{13}C NMR (125.7 MHz) spectrum of prosperin C (10) in CD_3COCD_3 .... S23
Figure S33. HSQC spectrum of prosperin C (10) S23
Figure S34. COSY spectrum of prosperin C (10) S24
Figure S35. H2BC spectrum of prosperin C (10) S24
Figure S36. HMBC spectrum of prosperin C (10) S25
Figure S37. NOESY spectrum of prosperin C (10) S25
Figure S38. ECD spectrum of prosperin C (10) in MeOH S26
Figure S39. IR spectrum of prosperin C (10) S26
Figure S40. UV spectrum of prosperin C (10) S26
Details about computational NMR calculations of prosperin A (8) in chloroform S27
Table S2. Experimental and computed ^{1}H NMR of prosperin A (8) in $CDCl_3$ S27
Table S3. Experimental and computed ^{13}C NMR of prosperin A (8) in $CDCl_3$ S28
Table S4. Comparison of ^{1}H and ^{13}C NMR of prosperin B (9) in CD_3COCD_3 .... S28
Figure S41. ECD calculations for prosperin B (9) S30
Figure S42. ECD calculations for alternative prosperin C (10) stereoisomers S30
Figure S43. NMR calculations for alternative prosperin C (10) stereoisomers S32

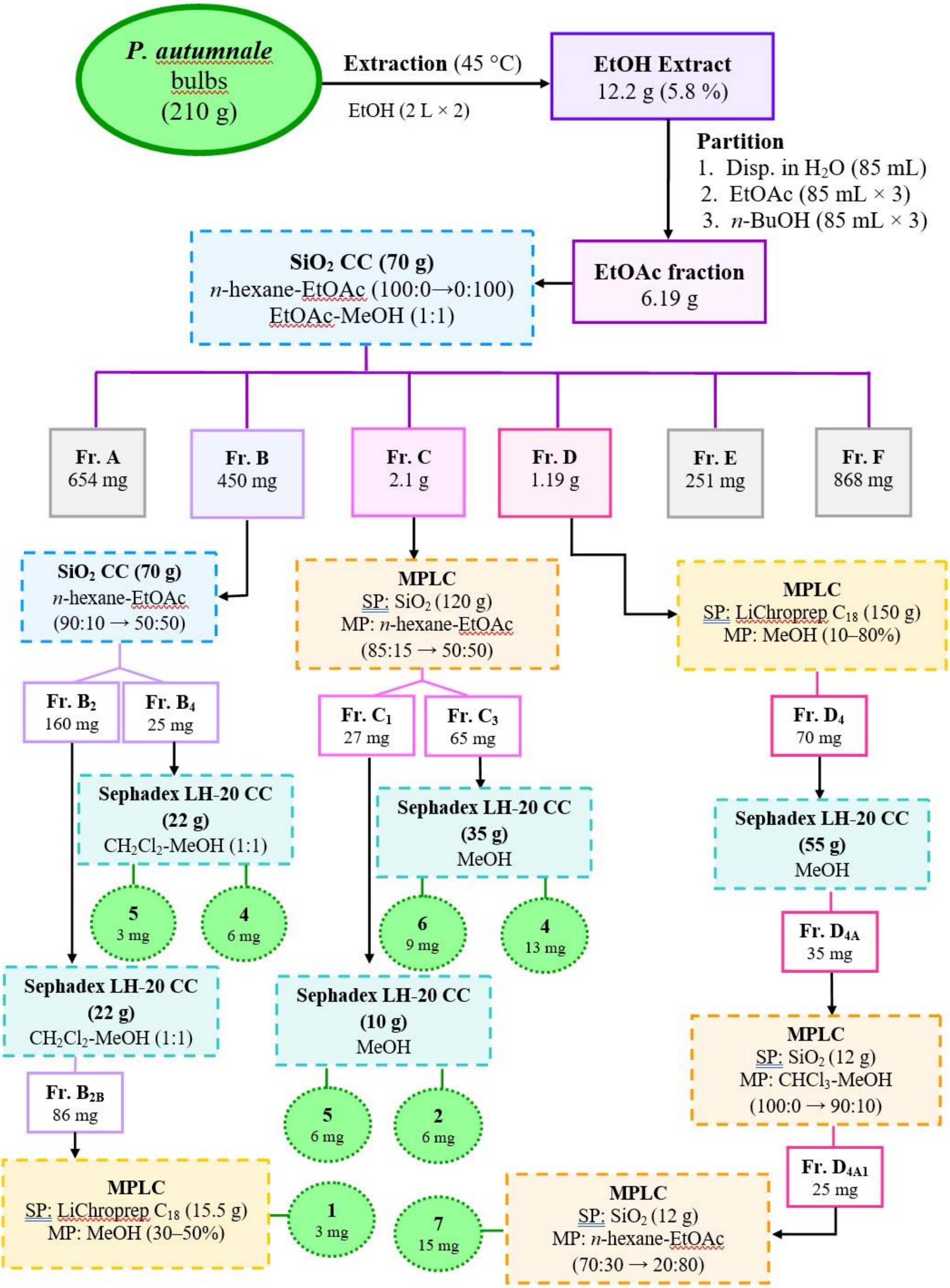

Scheme S1.1 Isolation of compounds from *Prospero autumnale*.

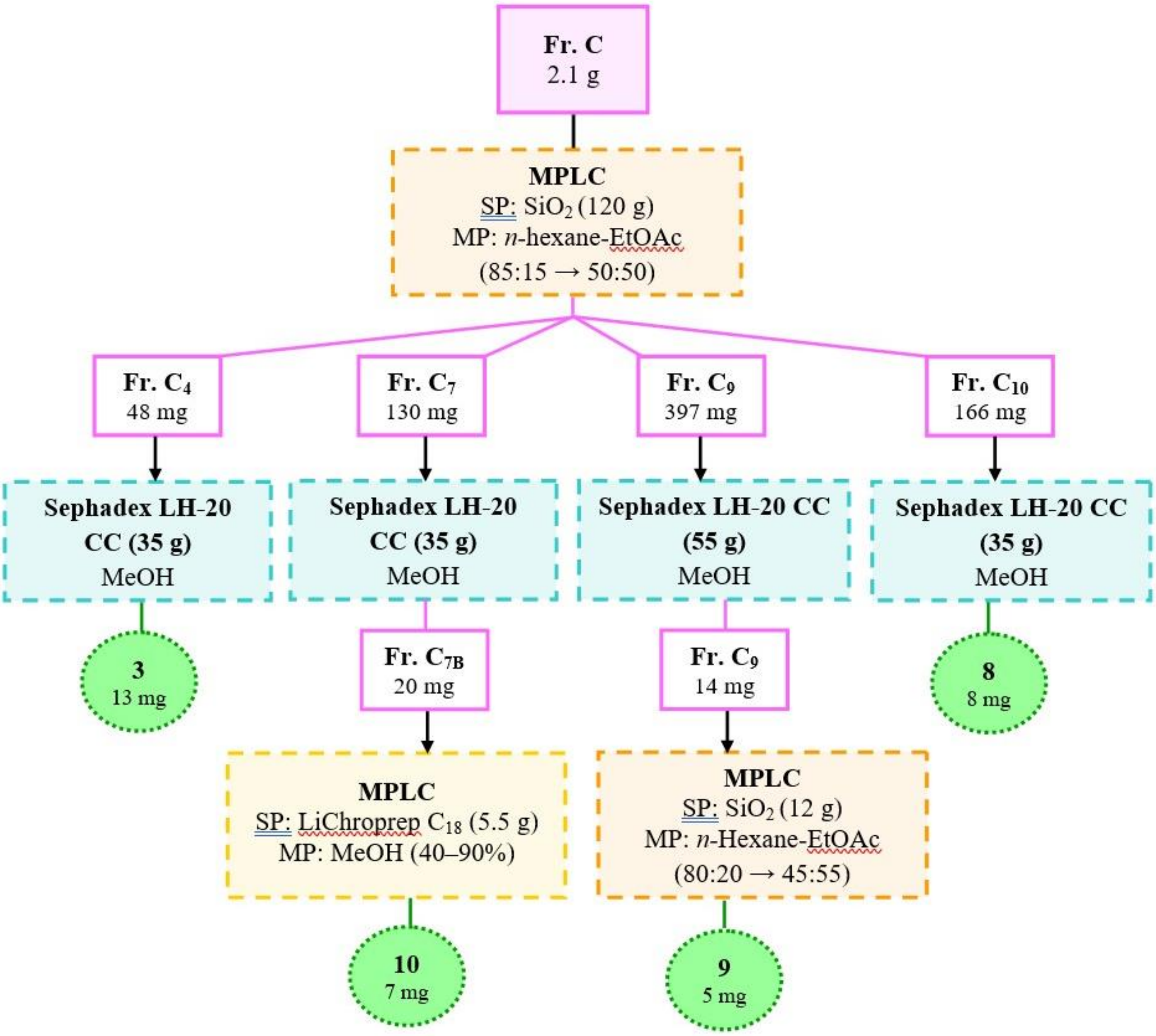

Scheme S1.2 Isolation of compounds from *Prospero autumnale.*

Table S1. Cytotoxic activities of the extract and fractions[a]

	Extract/Fraction $IC_{50} \pm SEM$ [μg/mL][a]		
**Cell Lines**	**EtOH extr.**	**EtOAc fraction**	**Doxorubicin**[b]
HCT116	59.99±2.76	22.01±5.13	0.86±0.14
LoVo	86.13±5.14	11.79±1.67	2.40±0.19
MCF7	81.52±2.10	16.92±1.08	2.32±0.28
MDA-MB-231	>100	20.13±1.34	2.33±0.33
PC3	15.11±1.64	19.22±1.01	0.88±0.08
DU145	69.93±1.16	34.04±1.49	5.93±0.38
HEP3B	26.72±2.02	22.23±0.99	1.10±0.07
HEPG2	56.15±8.73	27.34±2.84	4.50±0.27
L929	>100	21.52±1.97	>100

[a]IC_{50} for *n*-BuOH and H_2O fractions: >100 μg/mL. IC_{50} values were calculated from the cell growth inhibition curves obtained from the treatments with increasing concentrations of extracts or fractions for 48 h. Experiments were carried out in triplicate. [b] Positive control.

Figure S1. HRESIMS of prospestilbene (**1**)

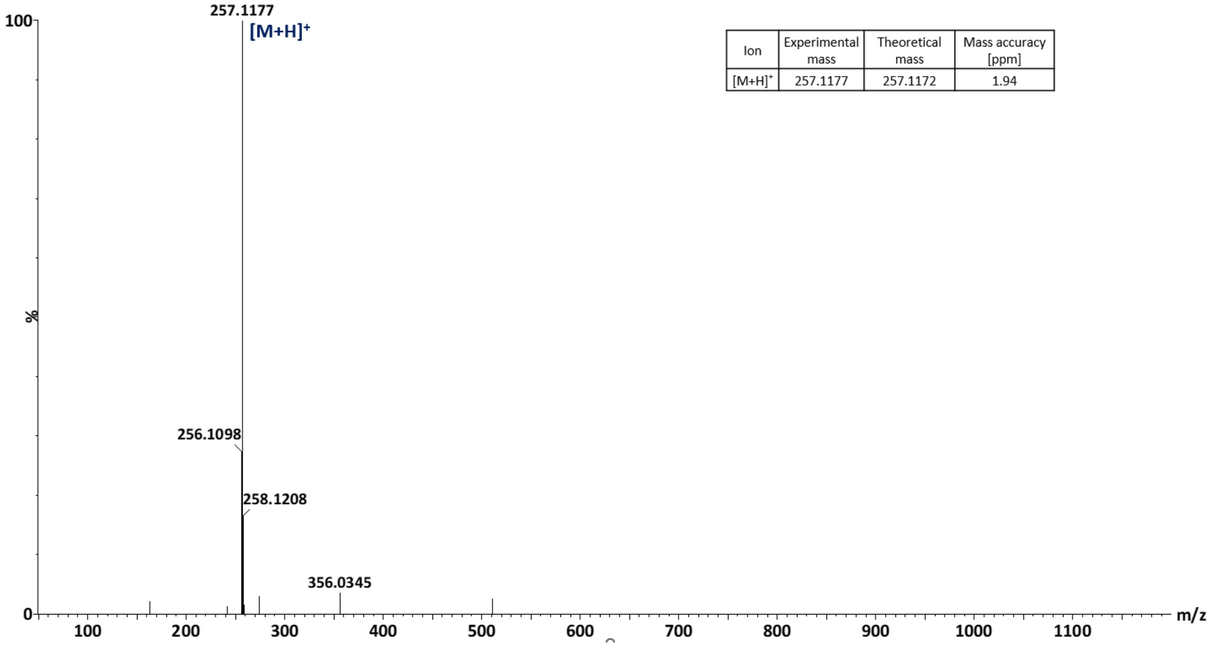

Ion	Experimental mass	Theoretical mass	Mass accuracy [ppm]
$[M+H]^+$	257.1177	257.1172	1.94

Figure S2. ^{1}H NMR (500 MHz) spectrum of prospestilbene (**1**) in CD_3OD

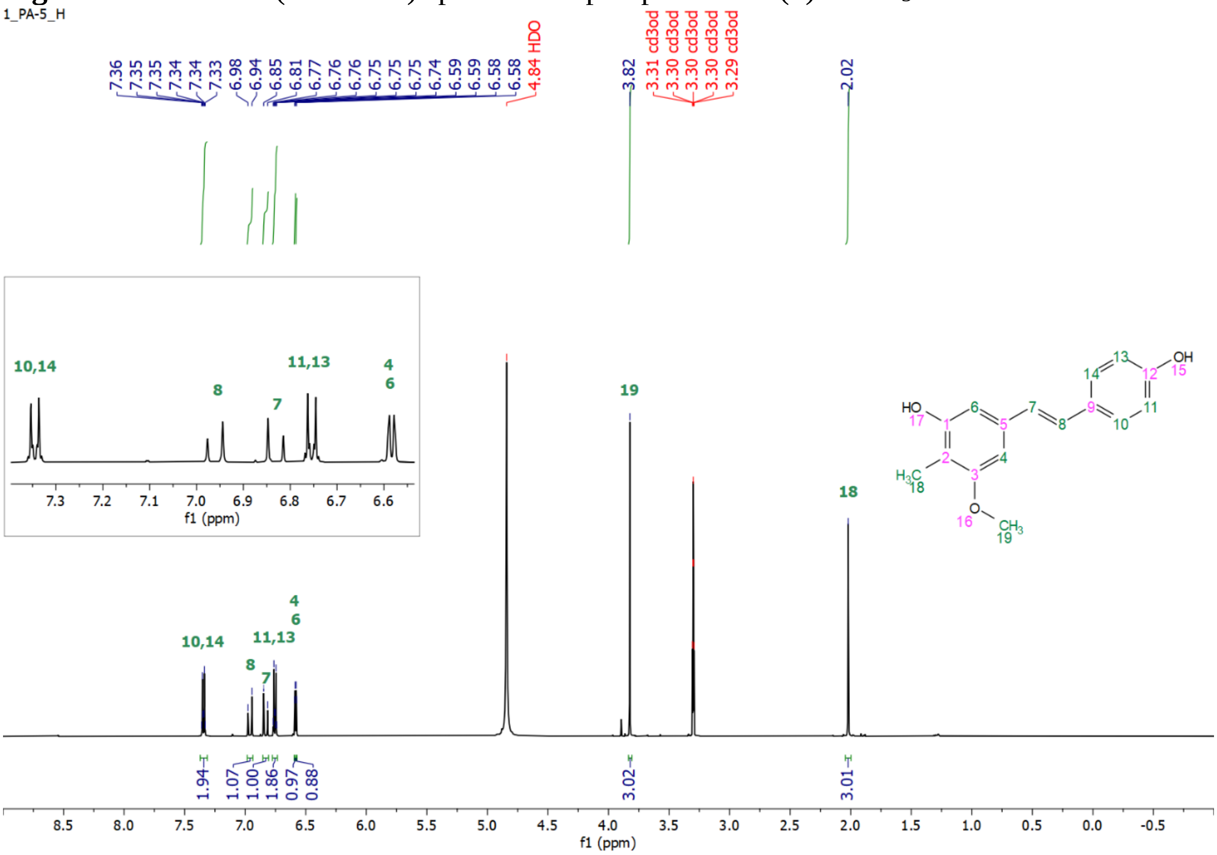

Figure S3. ^{13}C NMR (125.7 MHz) spectrum of prospestilbene (**1**) in CD_3OD

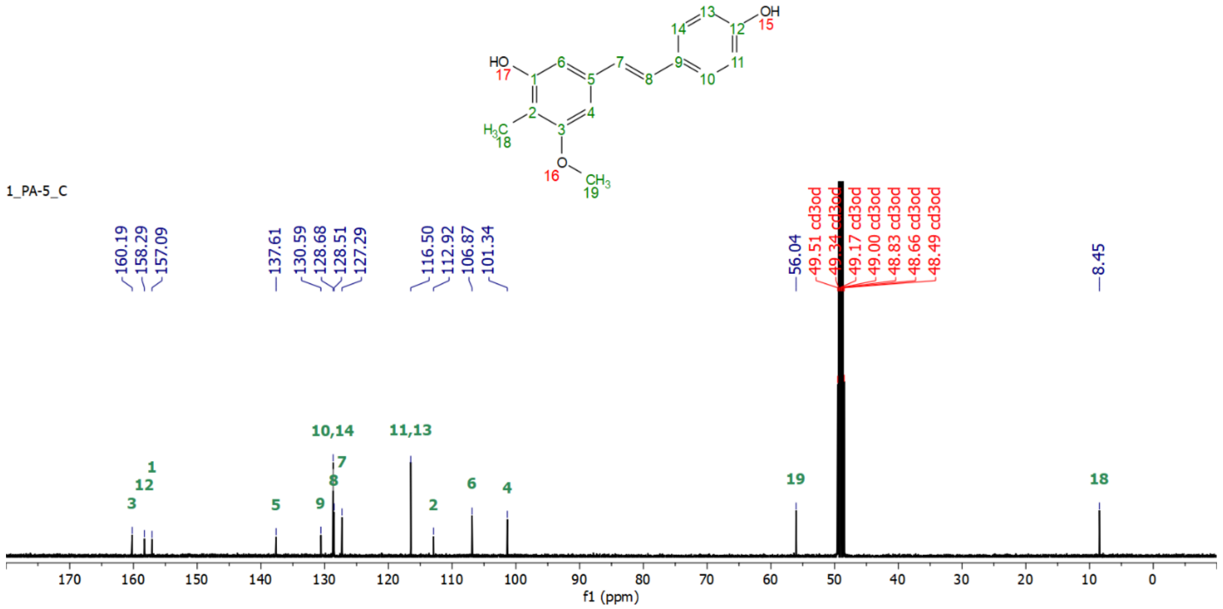

Figure S4. HSQC spectrum of prospestilbene (**1**)

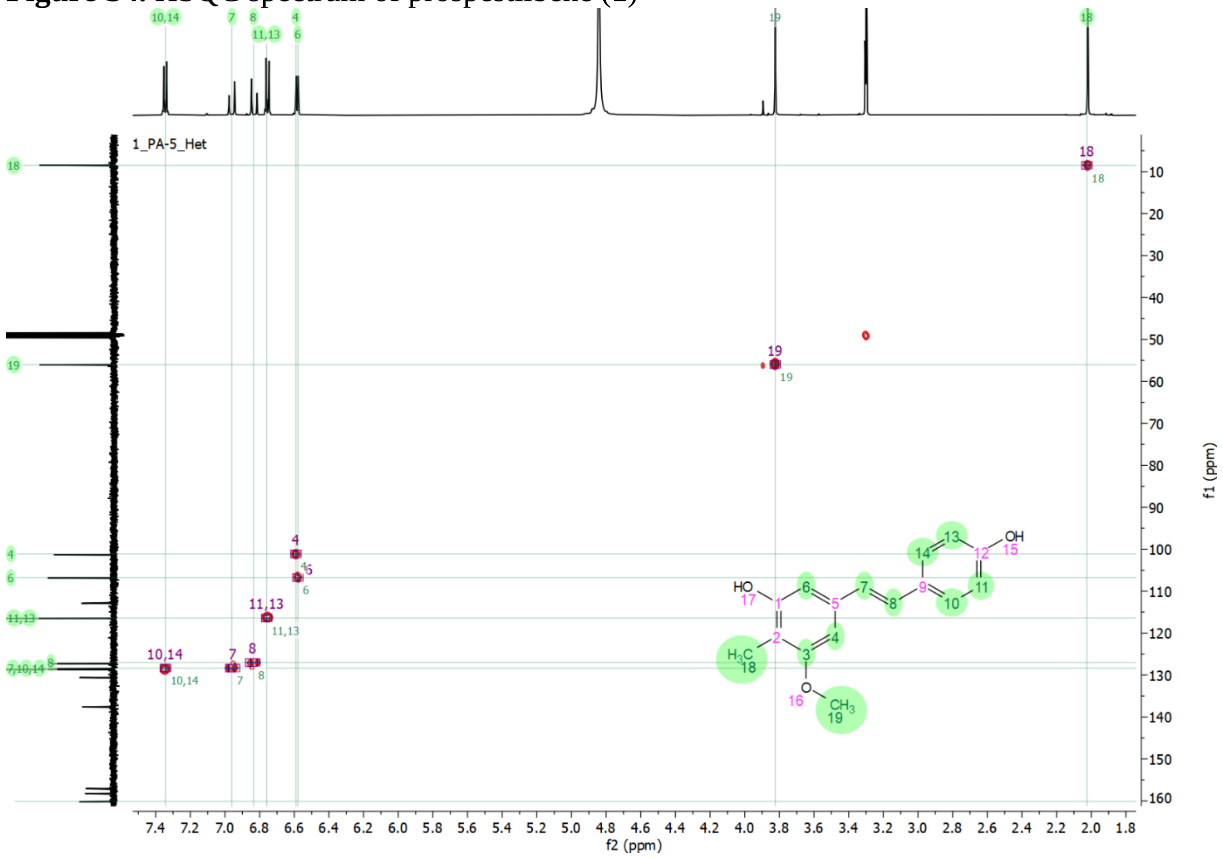

Figure S5. HMBC spectrum of prospestilbene (**1**)

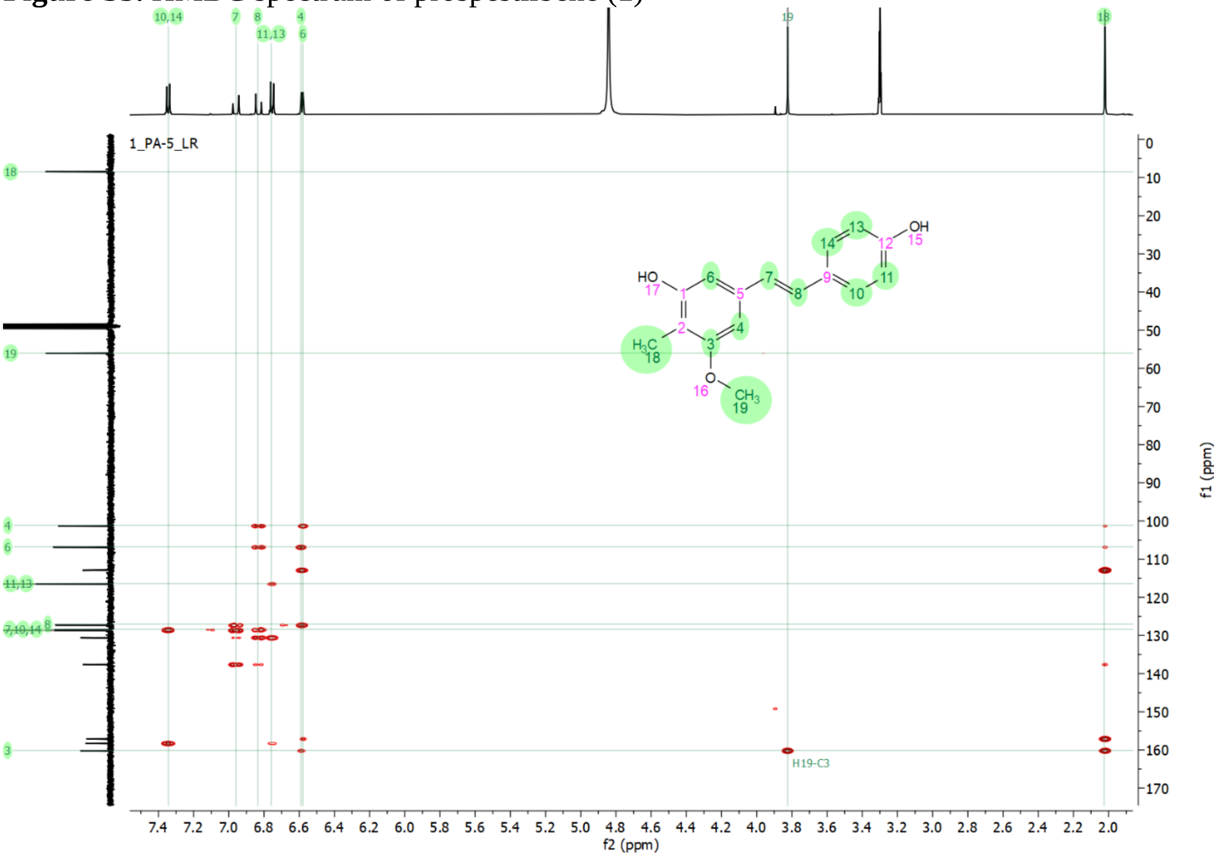

Figure S6. NOESY spectrum of prospestilbene (**1**)

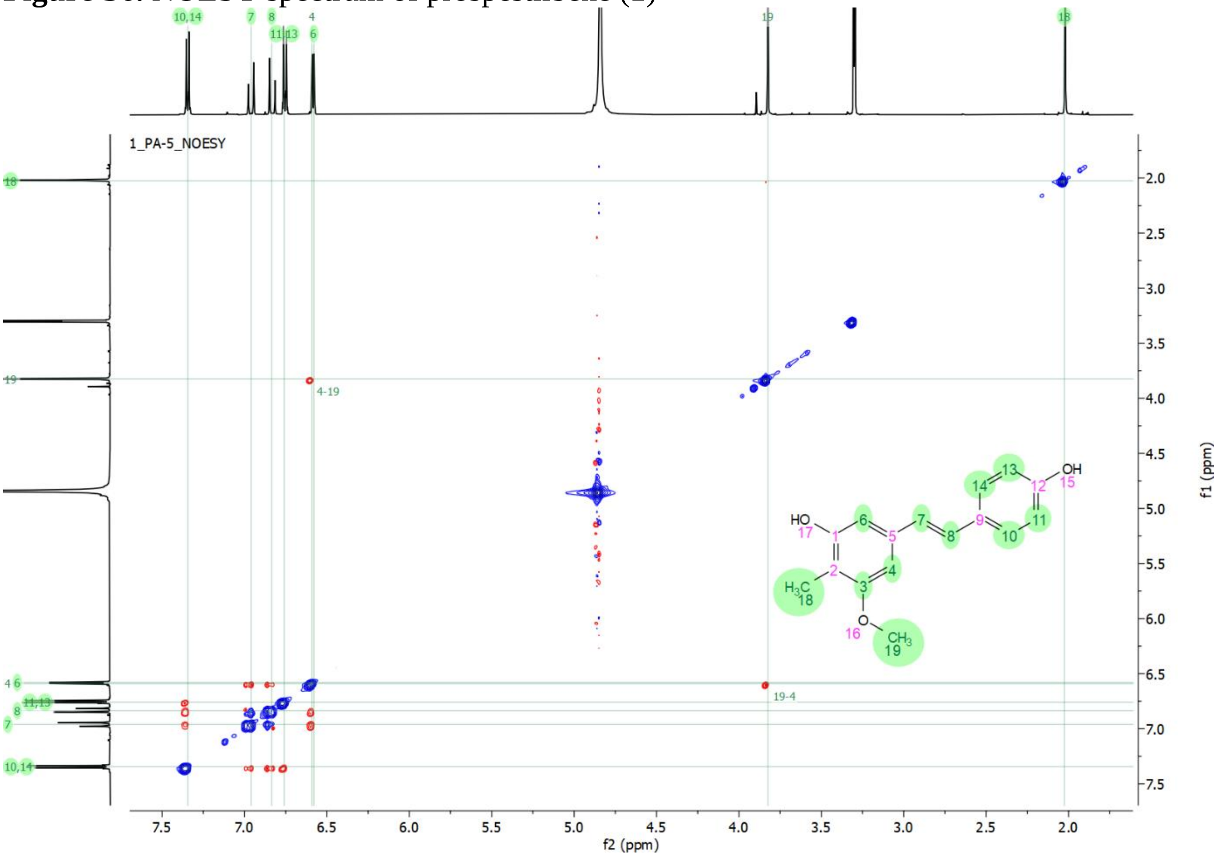

Figure S7. IR spectrum of prospestilbene (**1**)

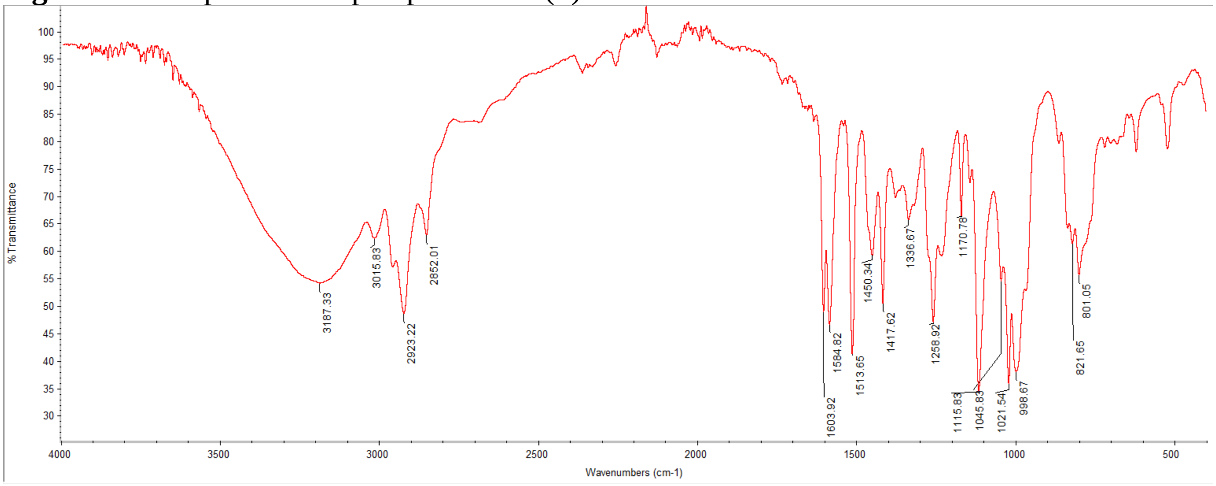

Figure S8. UV spectrum of prospestilbene (**1**)

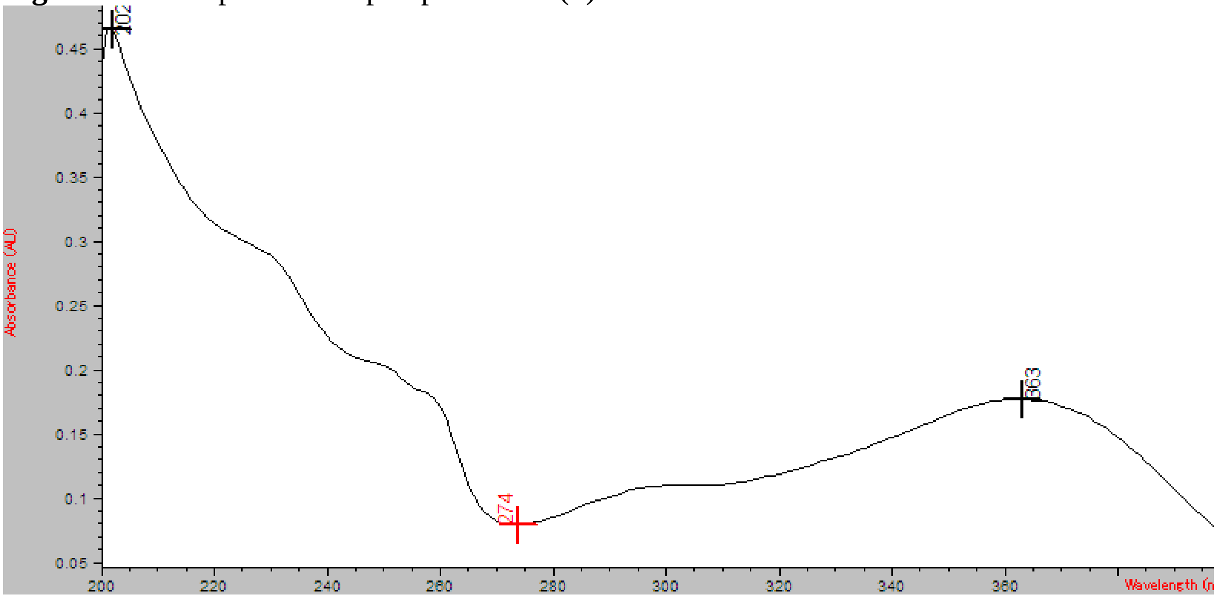

Figure S9. HRESIMS of prosperin A (**8**)

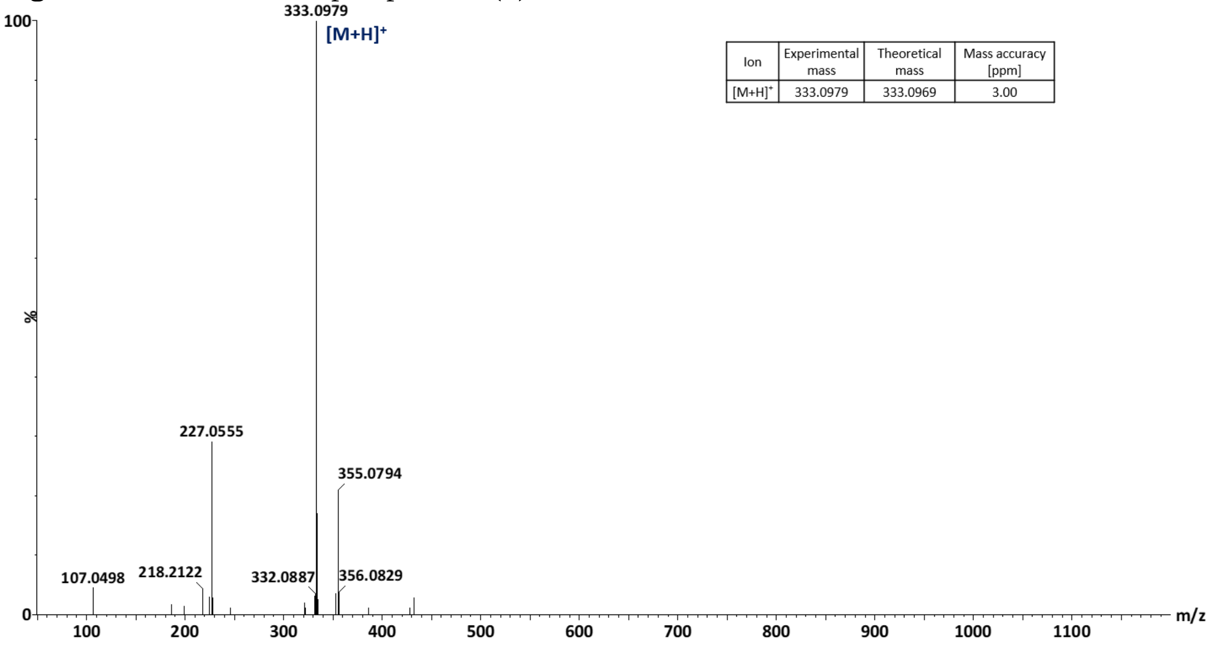

Ion	Experimental mass	Theoretical mass	Mass accuracy [ppm]
$[M+H]^+$	333.0979	333.0969	3.00

Figure S10. ^1H NMR (500 MHz) spectrum of prosperin A (**8**) in CD_3COCD_3

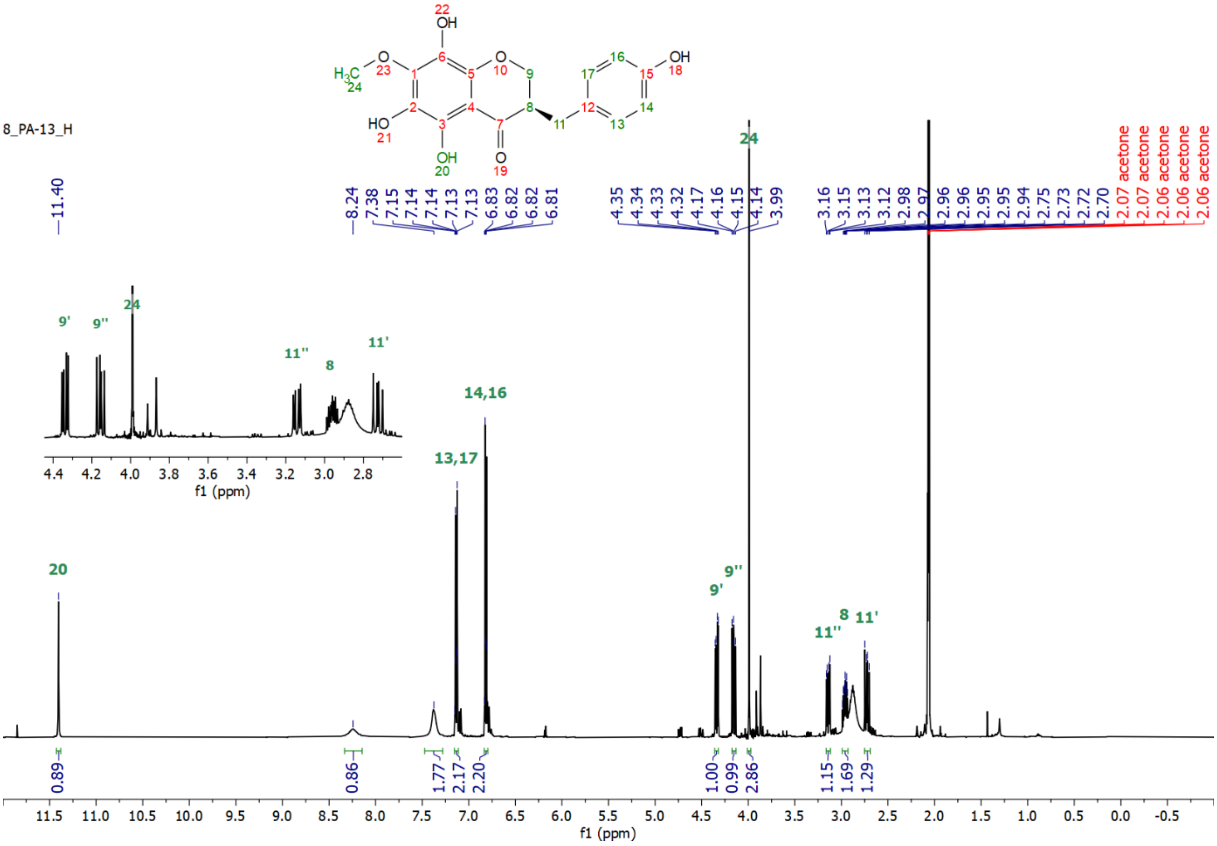

Figure S11. ^{13}C NMR (125.7 MHz) spectrum of prosperin A (**8**) in CD_3COCD_3

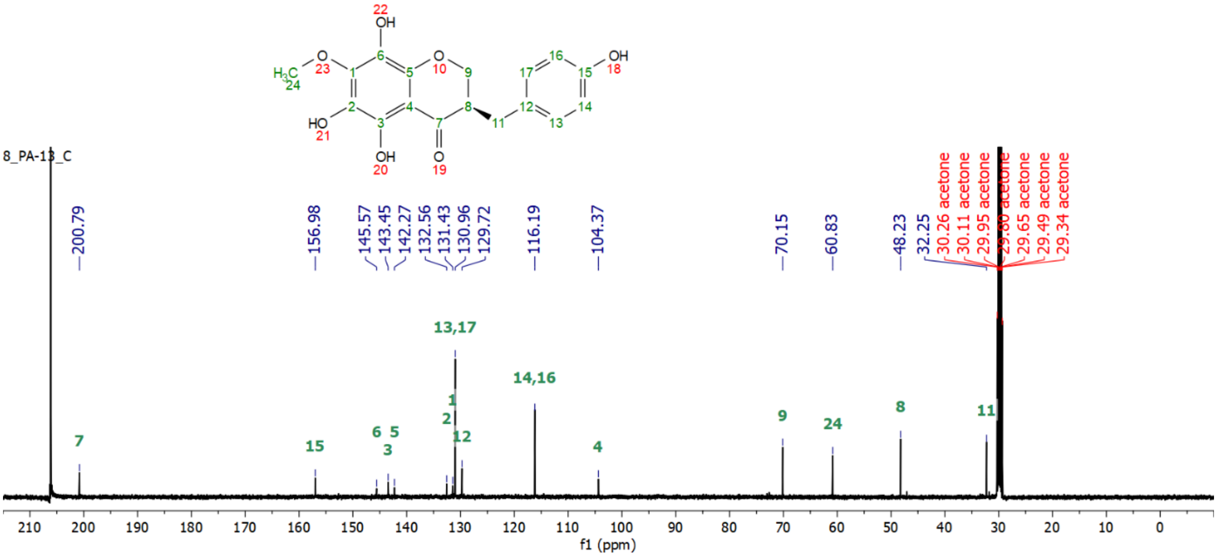

Figure S12. HSQC spectrum of prosperin A (**8**)

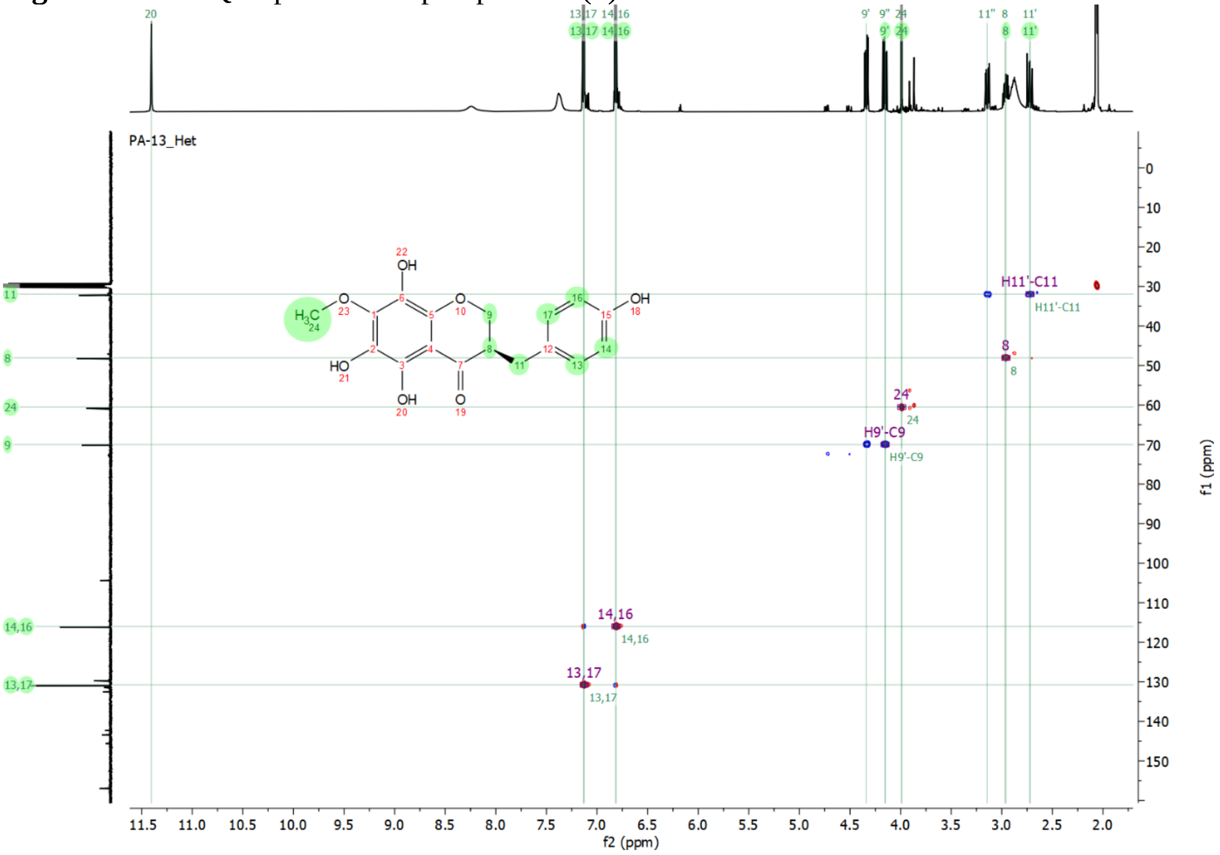

Figure S13. HMBC spectrum of prosperin A (**8**)

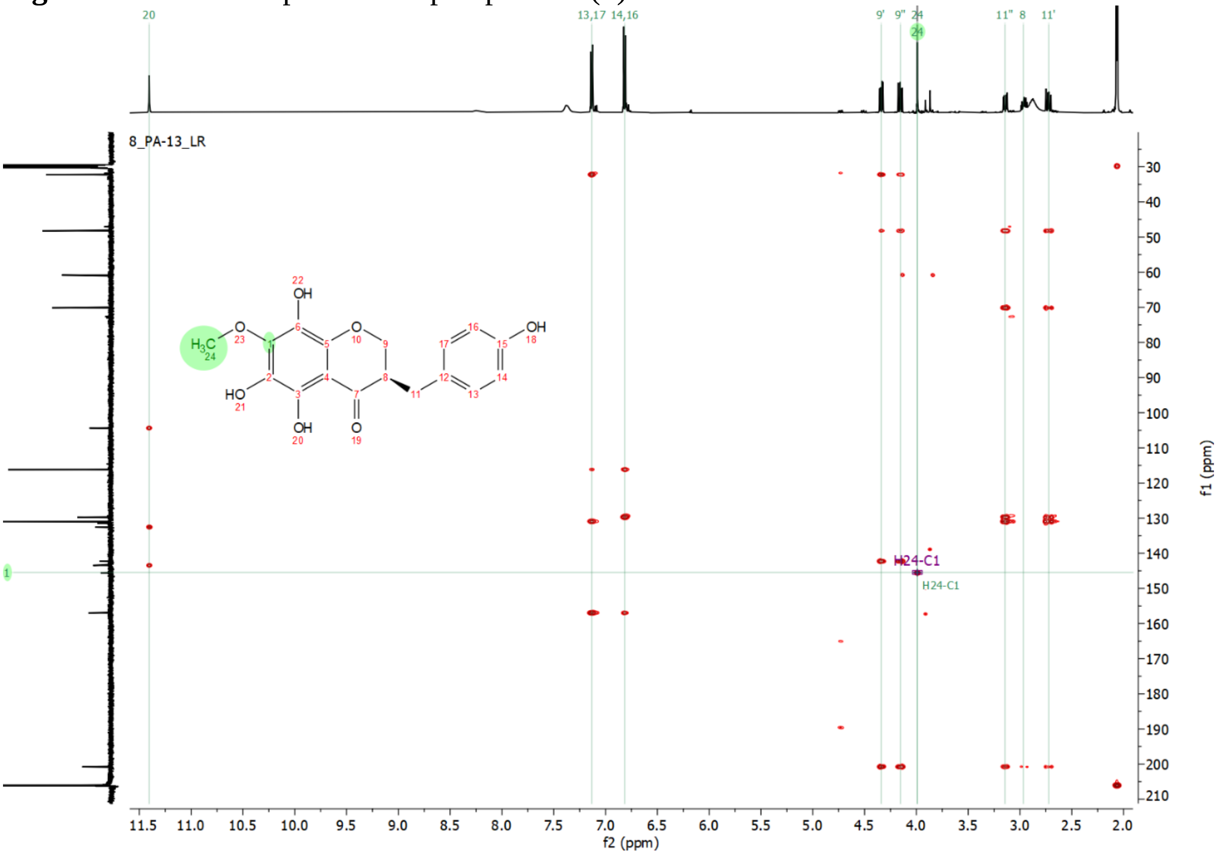

Figure S14. ROESY spectrum of prosperin A (**8**)

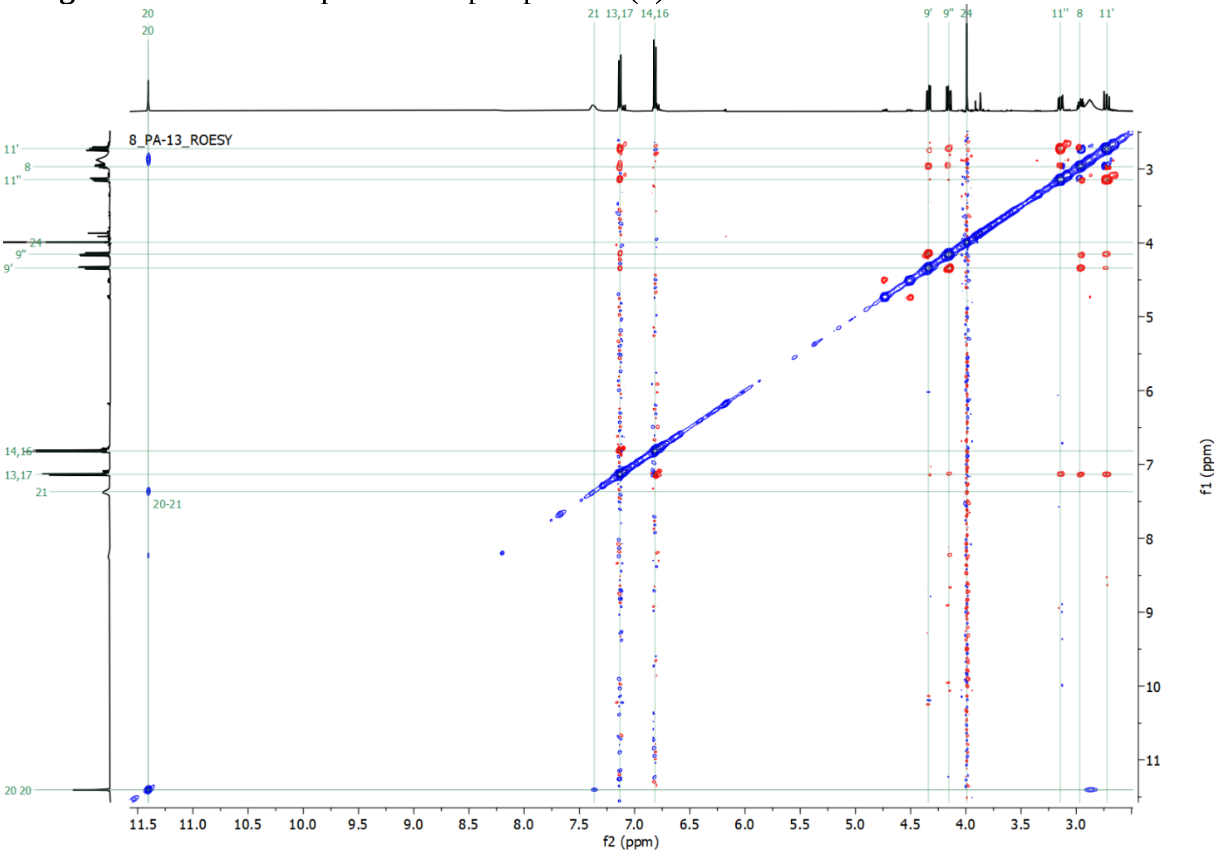

Figure S15. ^{1}H NMR (600 MHz) spectrum of prosperin A (**8**) in $CDCl_3$

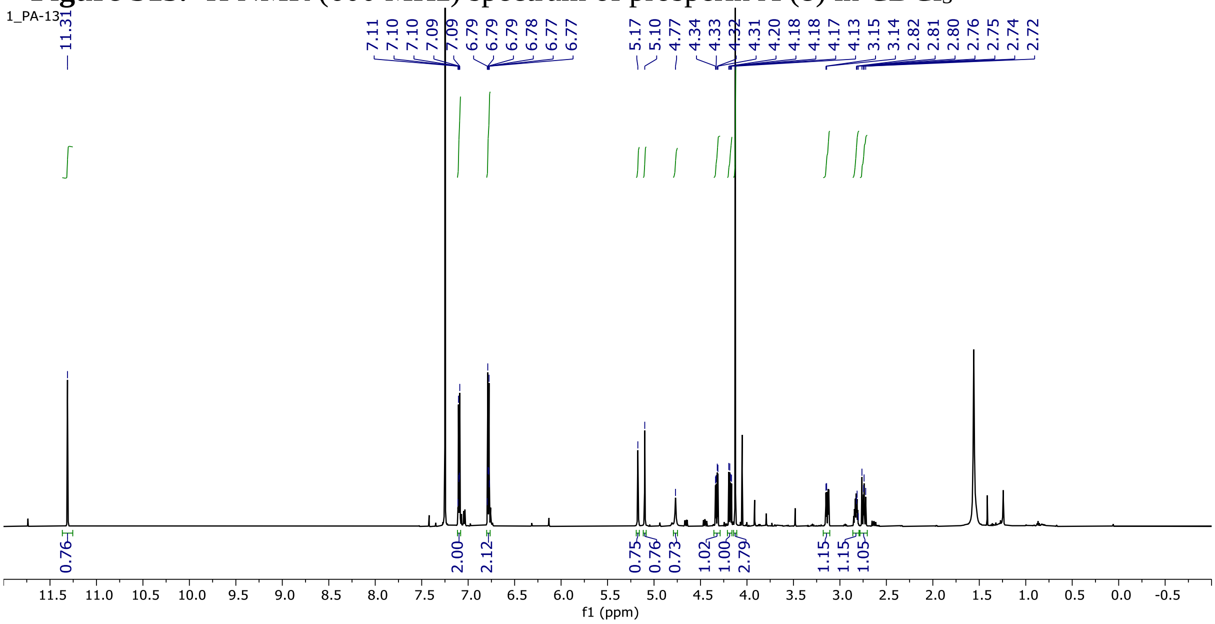

Figure S16. ^{13}C NMR (150 MHz) spectrum of prosperin A (**8**) in $CDCl_3$

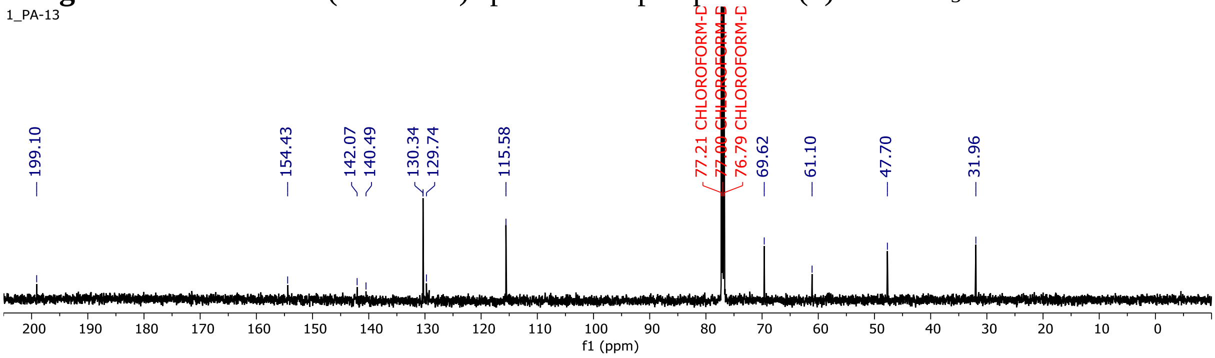

Figure S17. ECD spectrum of prosperin A (**8**) in MeOH

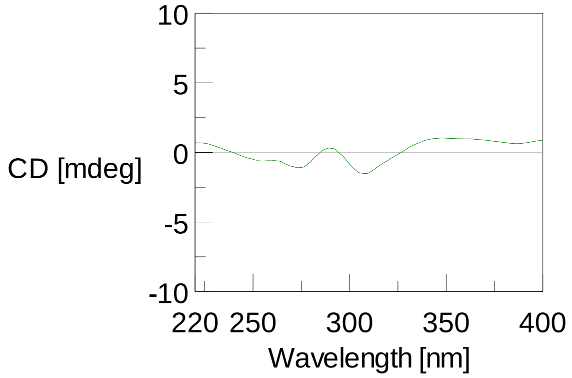

ECD (c 0.2 mg/ml, MeOH)

$\Delta\varepsilon_{273} = -0.55$ $[\theta]_{273} = -1826$

$\Delta\varepsilon_{308} = -0.76$ $[\theta]_{308} = -2507$

Figure S18. IR spectrum of prosperin A (**8**)

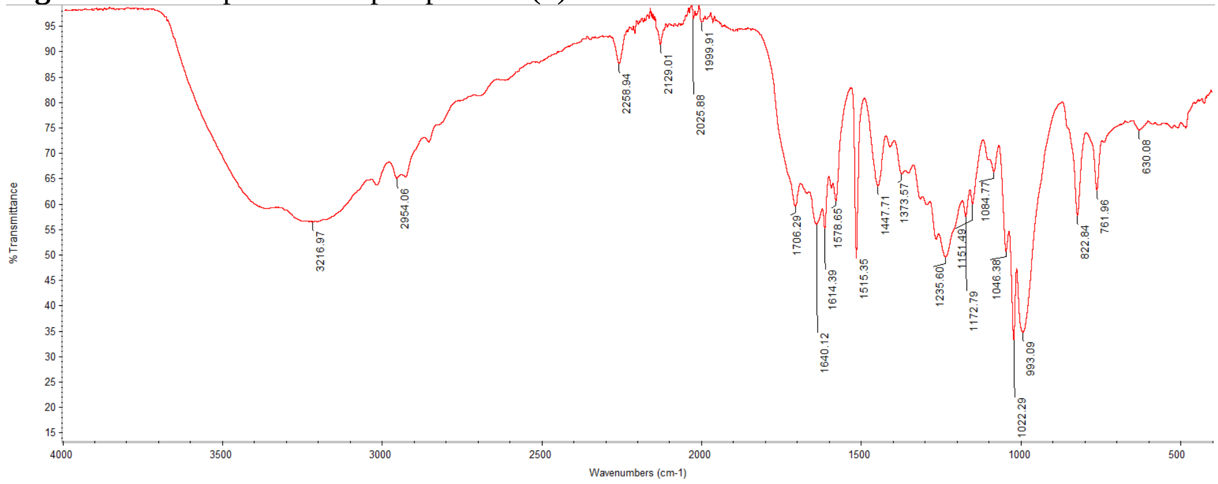

Figure S19. UV spectrum of prosperin A (**8**)

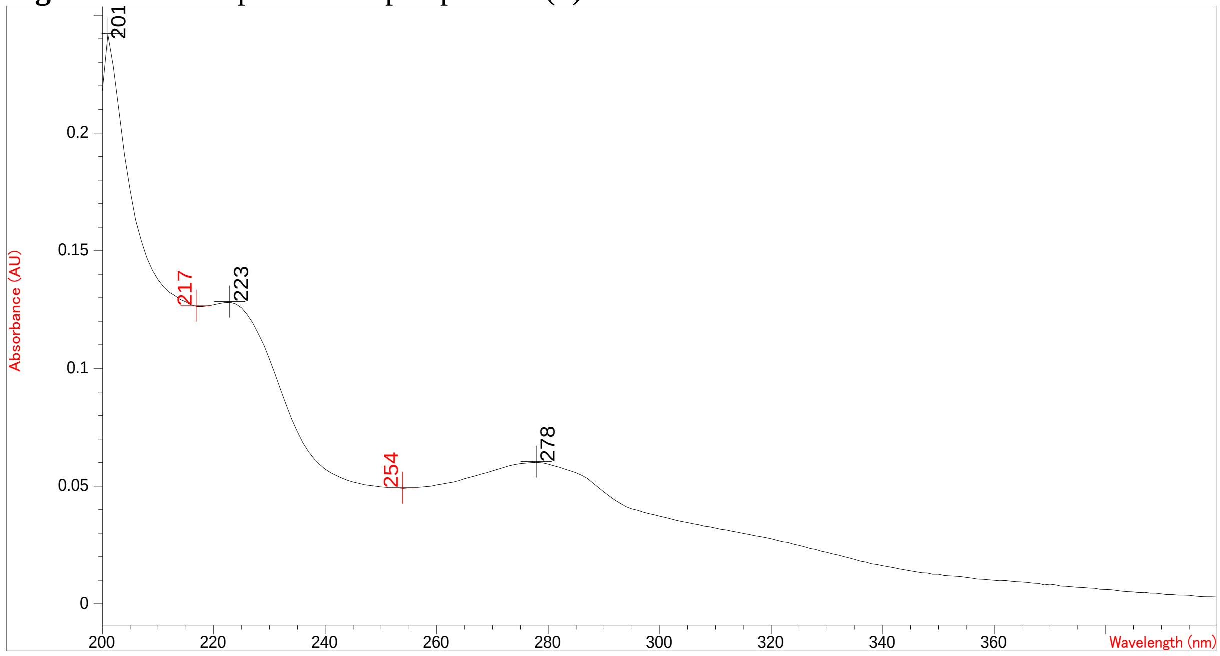

Figure S20. HRESIMS of prosperin B (**9**)

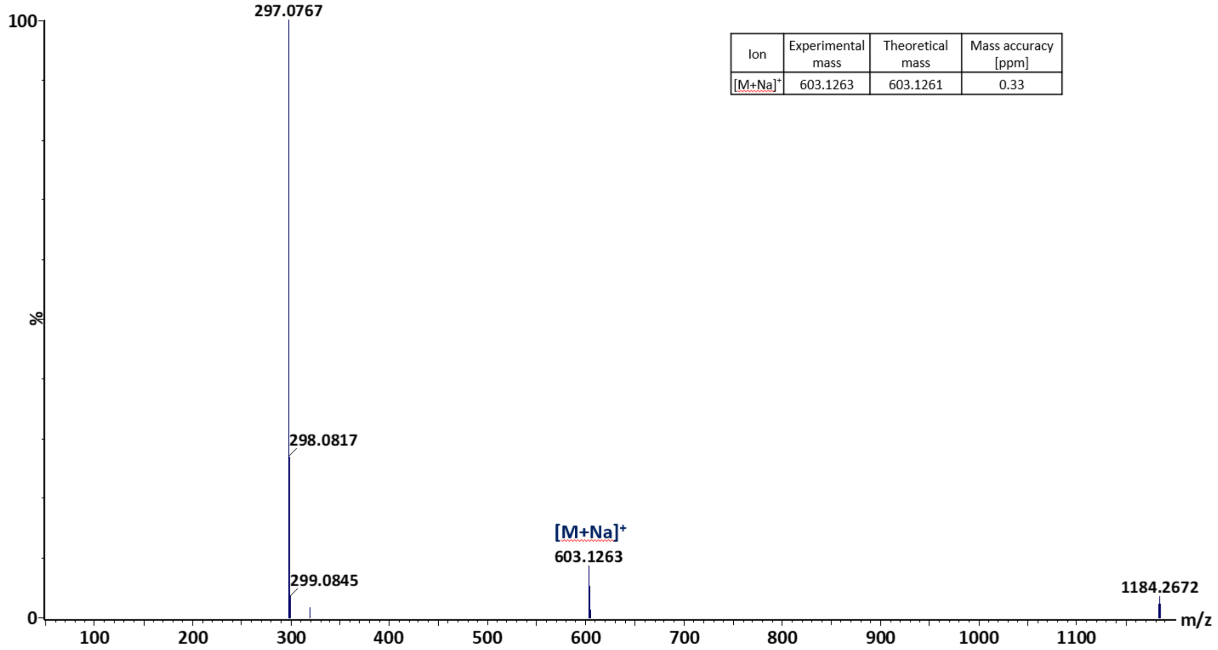

Figure S21. ^{1}H NMR (500 MHz) spectrum of prosperin B (**9**) in CD_3COCD_3

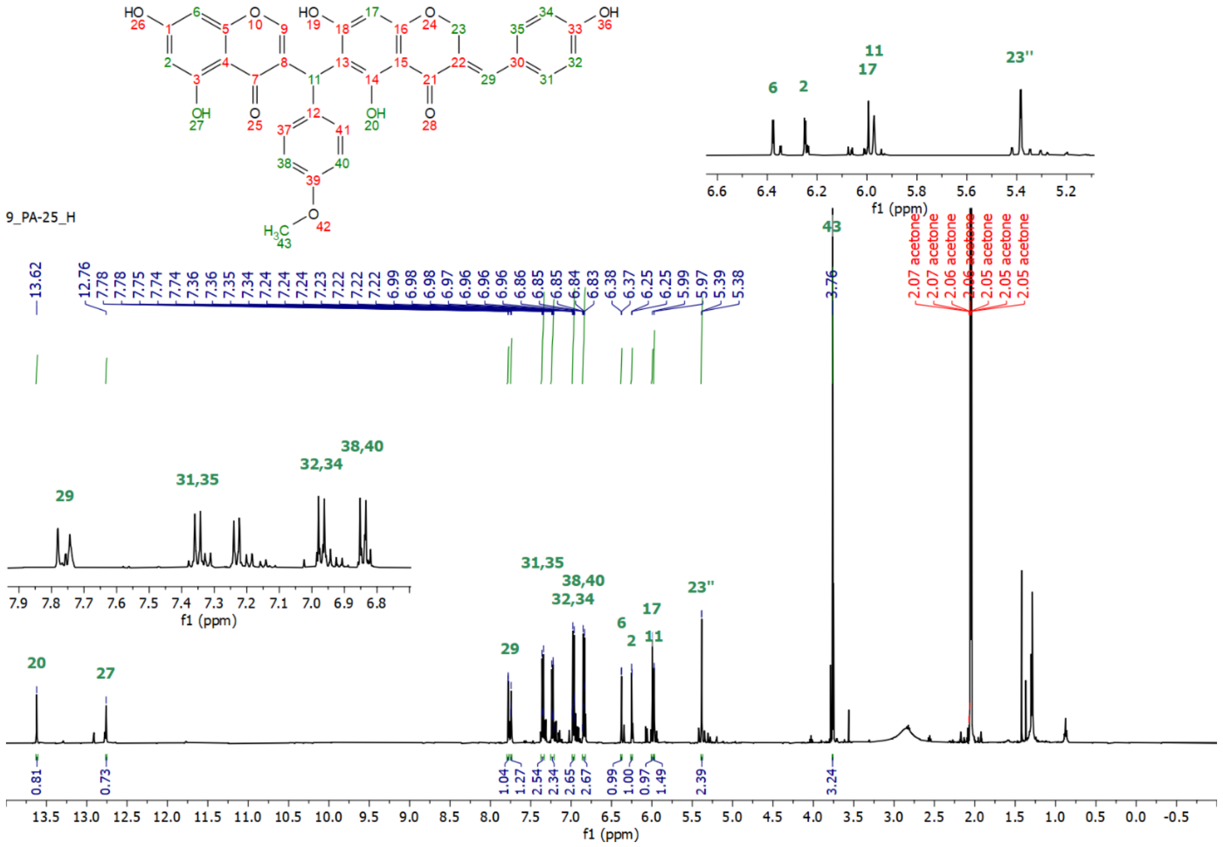

Figure S22. ^{13}C NMR (125.7 MHz) spectrum of prosperin B (**9**) in CD_3COCD_3

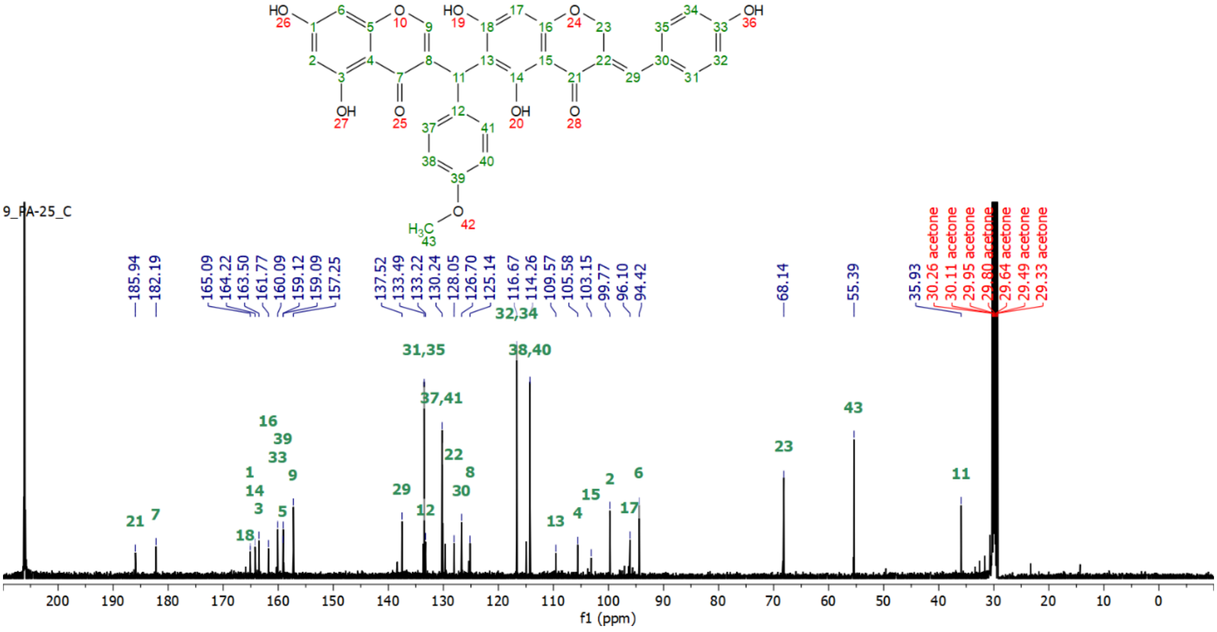

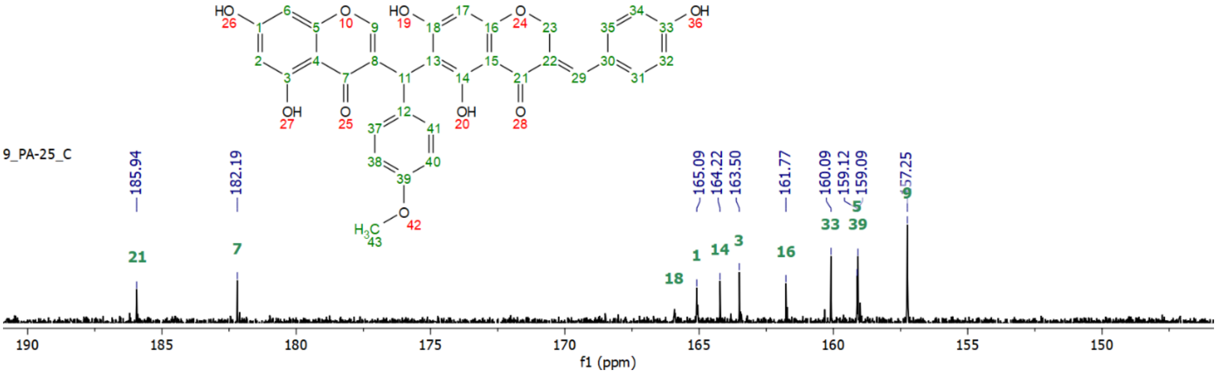

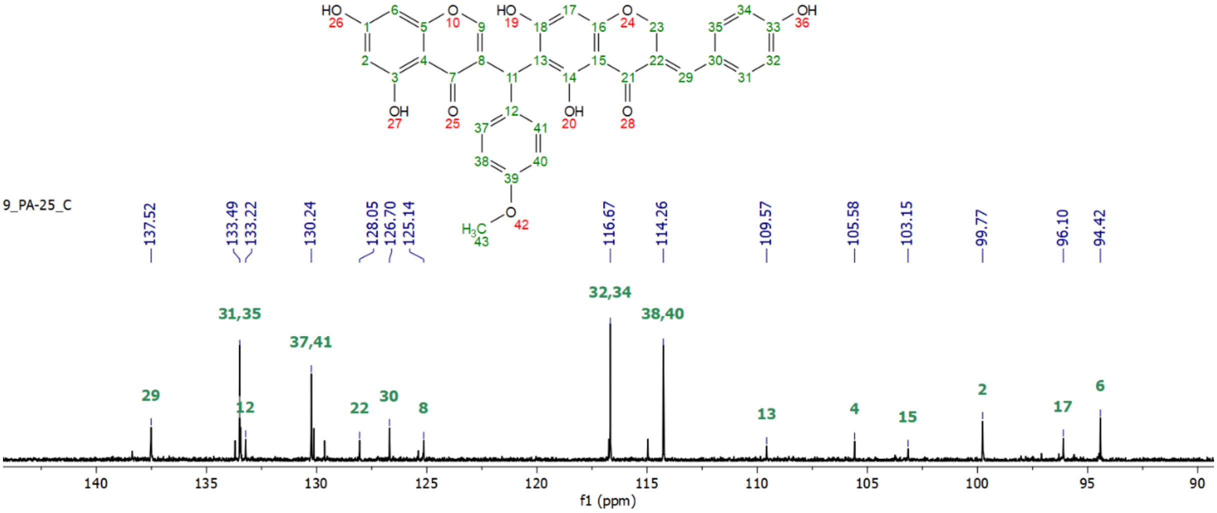

Figure S23. HSQC spectrum of prosperin B (**9**)

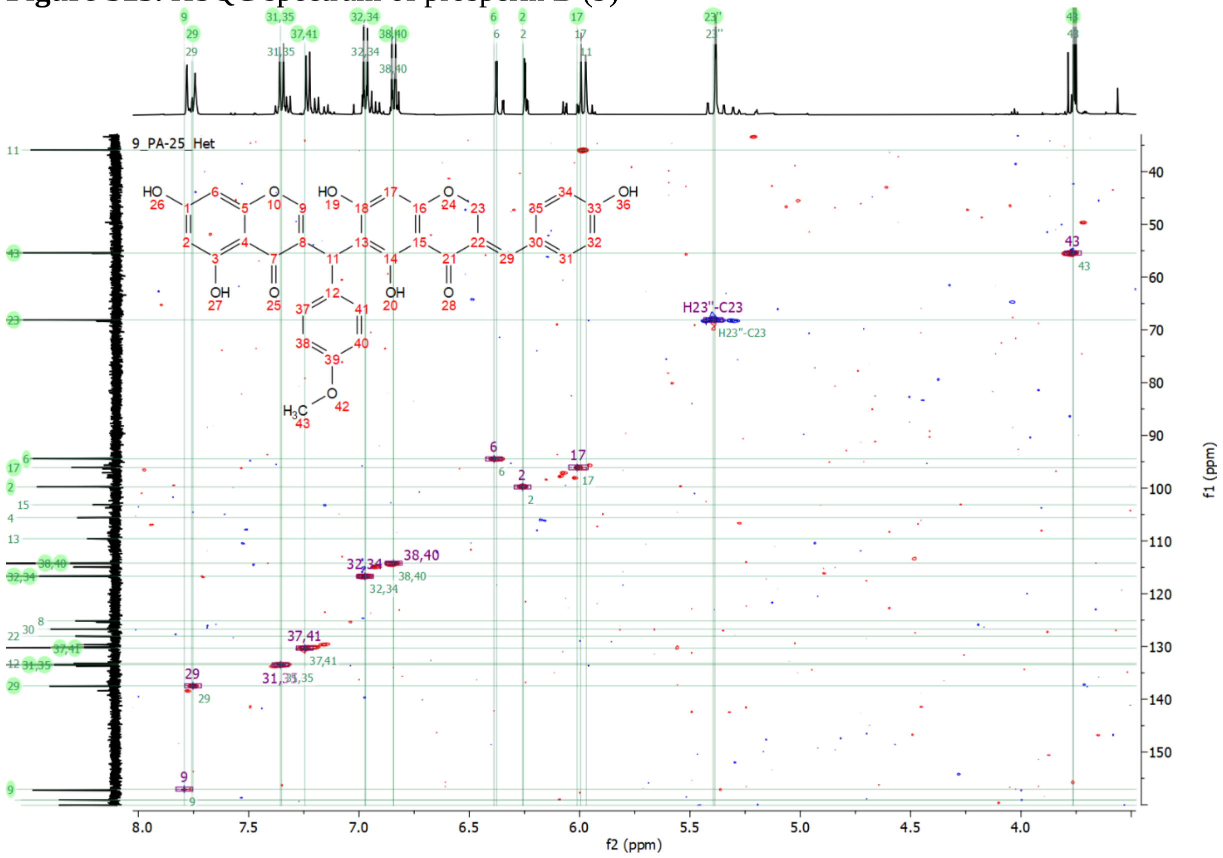

Figure S24. COSY spectrum of prosperin B (**9**)

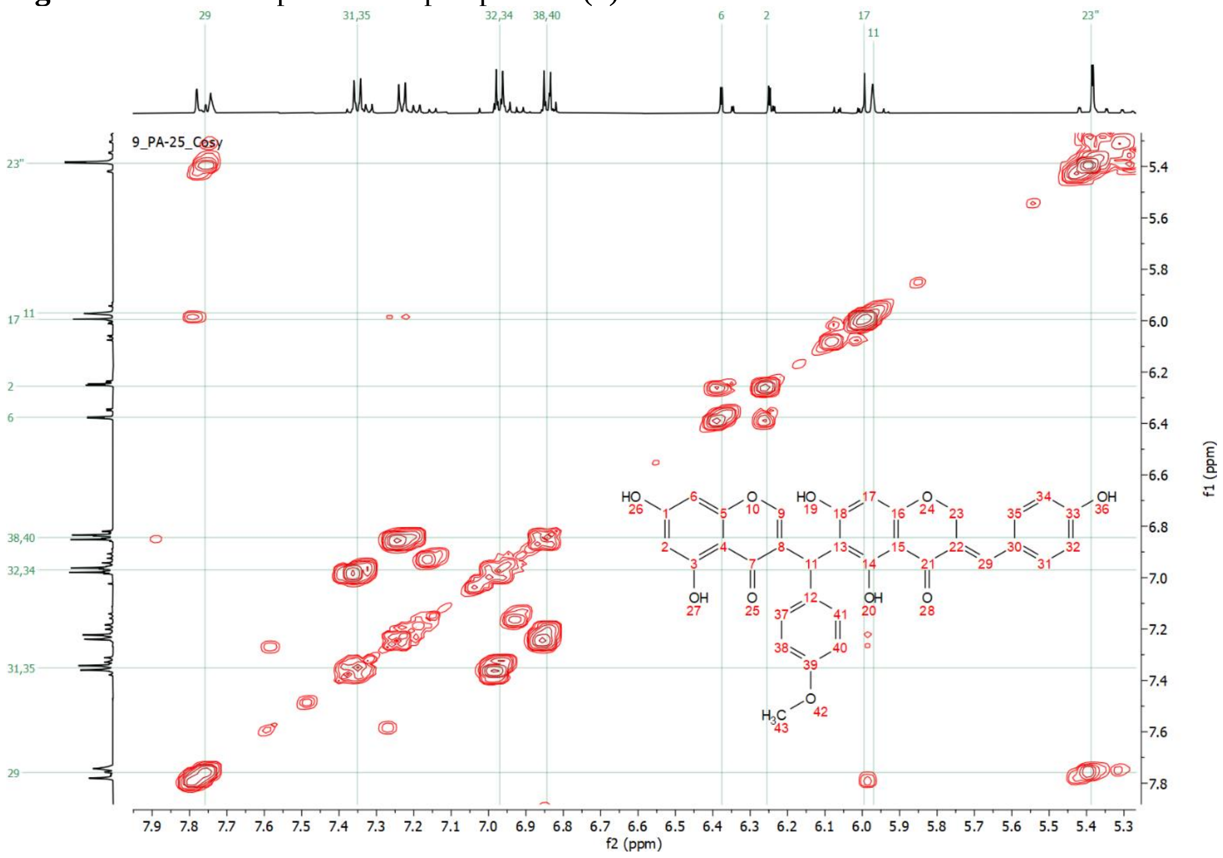

Figure S25. HMBC spectrum of prosperin B (**9**)

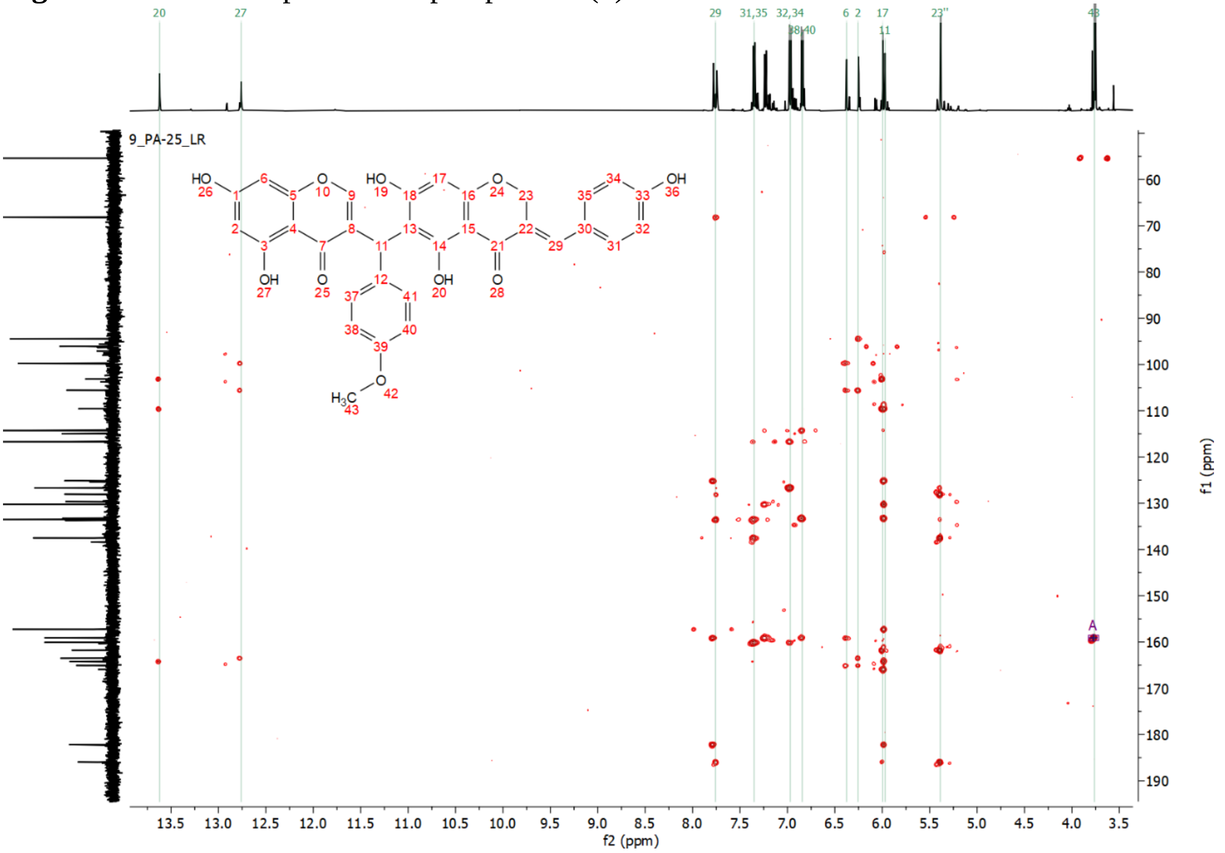

Figure S26. NOESY spectrum of prosperin B (**9**)

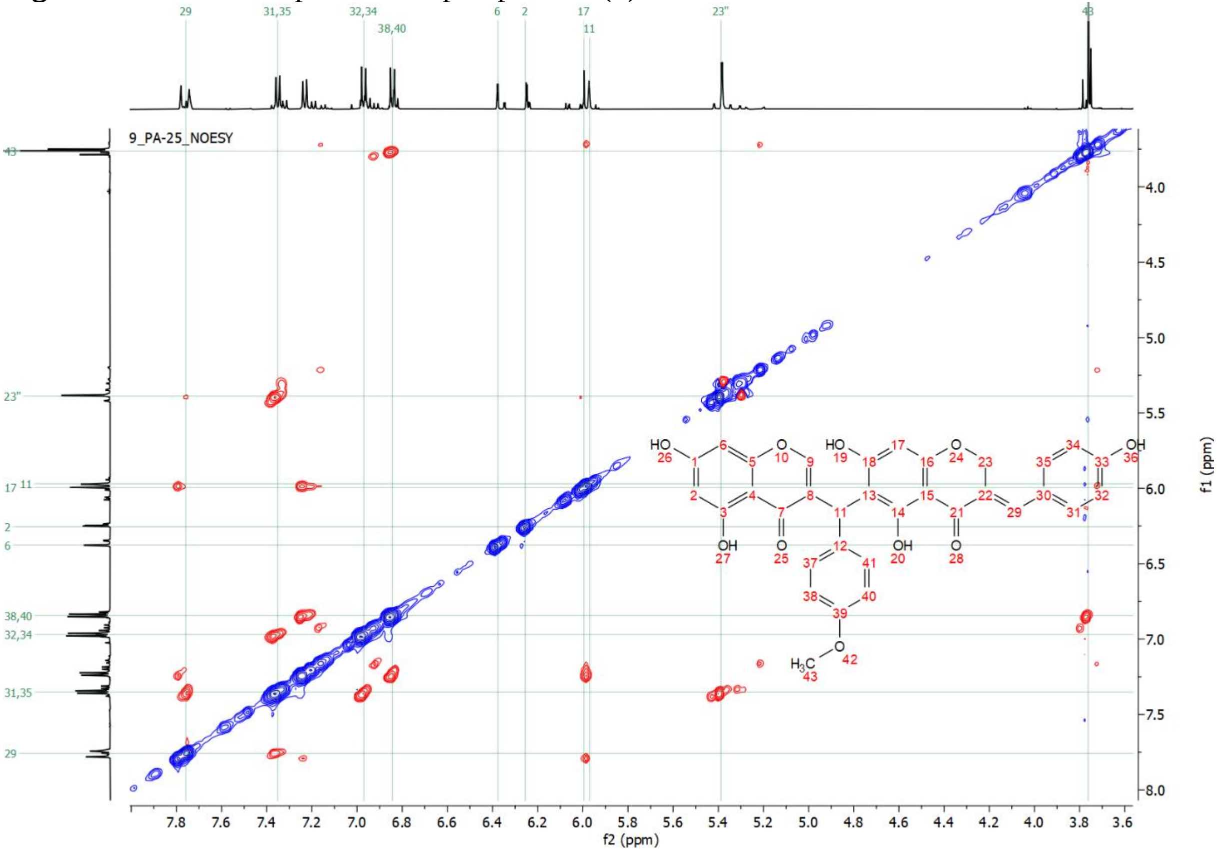

Figure S27. ECD spectrum of prosperin B (**9**) in MeOH

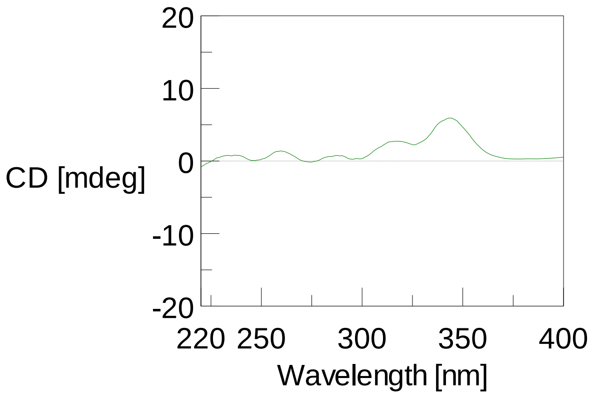

ECD (c 0.2 mg/ml, MeOH)

$\Delta\varepsilon_{237}$ = +0.69	$[\theta]_{237}$ = +2291
$\Delta\varepsilon_{260}$ = +1.20	$[\theta]_{260}$ = +3974
$\Delta\varepsilon_{287}$ = +0.66	$[\theta]_{287}$ = +2175
$\Delta\varepsilon_{317}$ = +2.38	$[\theta]_{317}$ = +7861
$\Delta\varepsilon_{343}$ = +5.20	$[\theta]_{343}$ = +17143

Figure S28. IR spectrum of prosperin B (**9**)

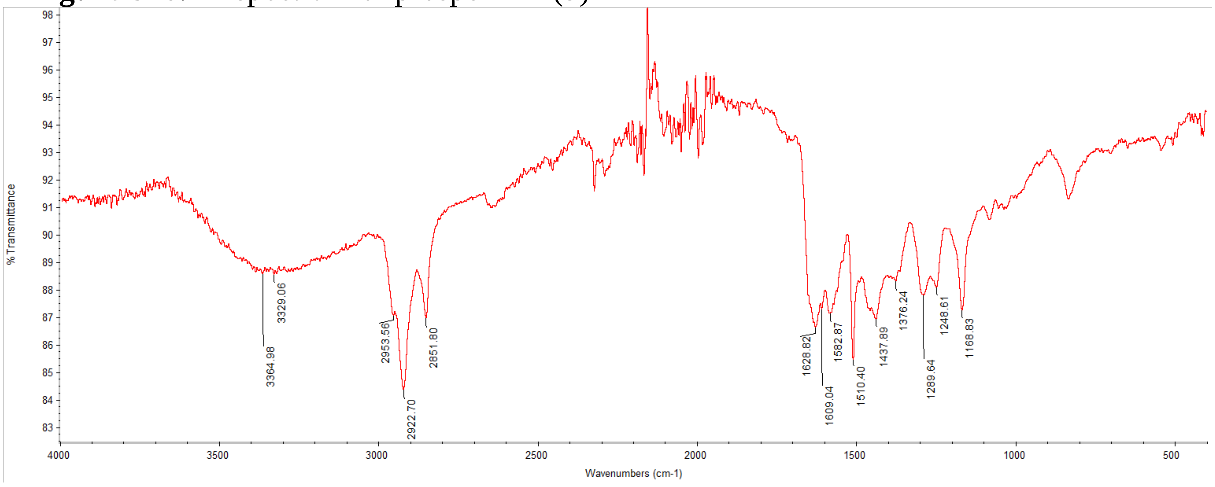

Figure S29. UV spectrum of prosperin B (**9**)

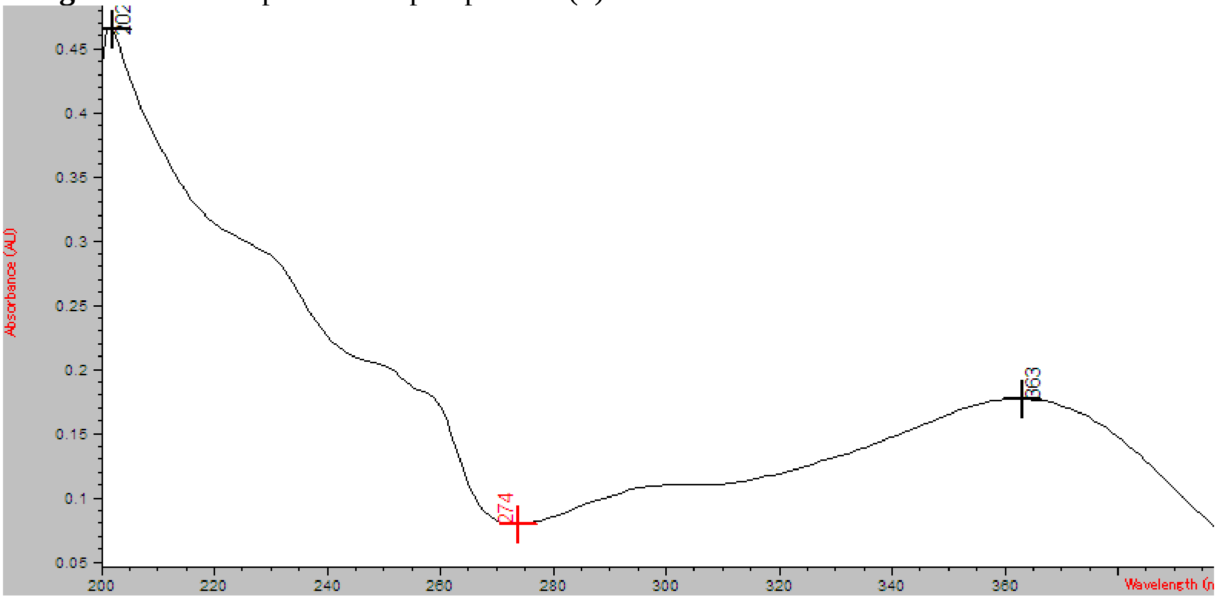

Figure S30. HRESIMS of prosperin C (**10**)

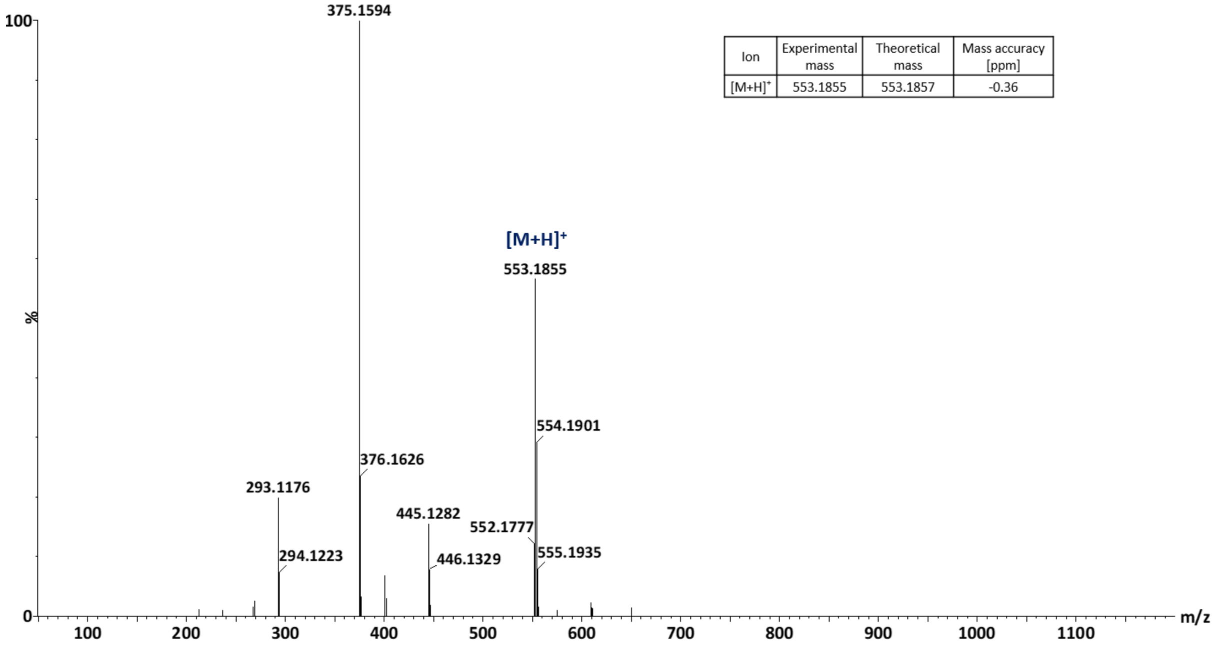

Ion	Experimental mass	Theoretical mass	Mass accuracy [ppm]
$[M+H]^+$	553.1855	553.1857	-0.36

Figure S31. ^{1}H NMR (500 MHz) spectrum of prosperin C (**10**) in CD_3COCD_3

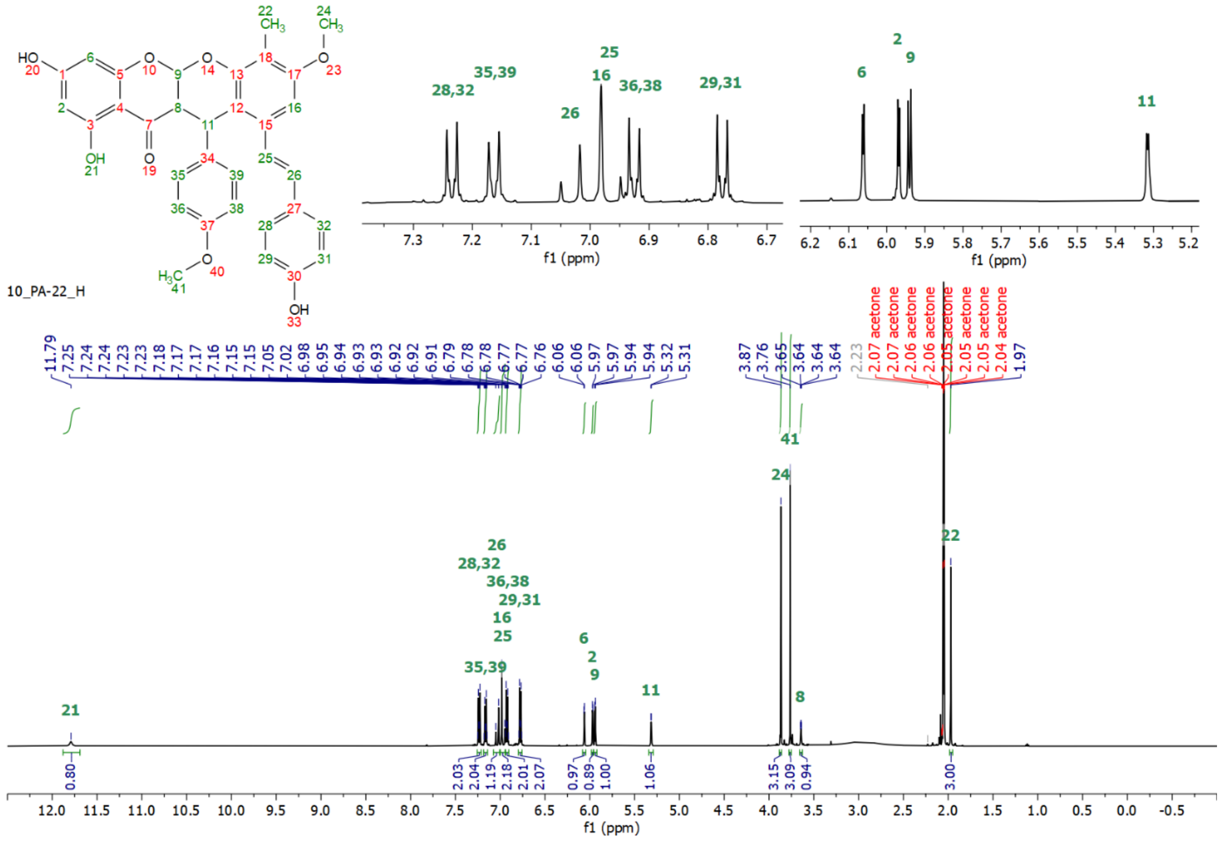

Figure S32. ^{13}C NMR (125.7 MHz) spectrum of prosperin C (**10**) in CD_3COCD_3

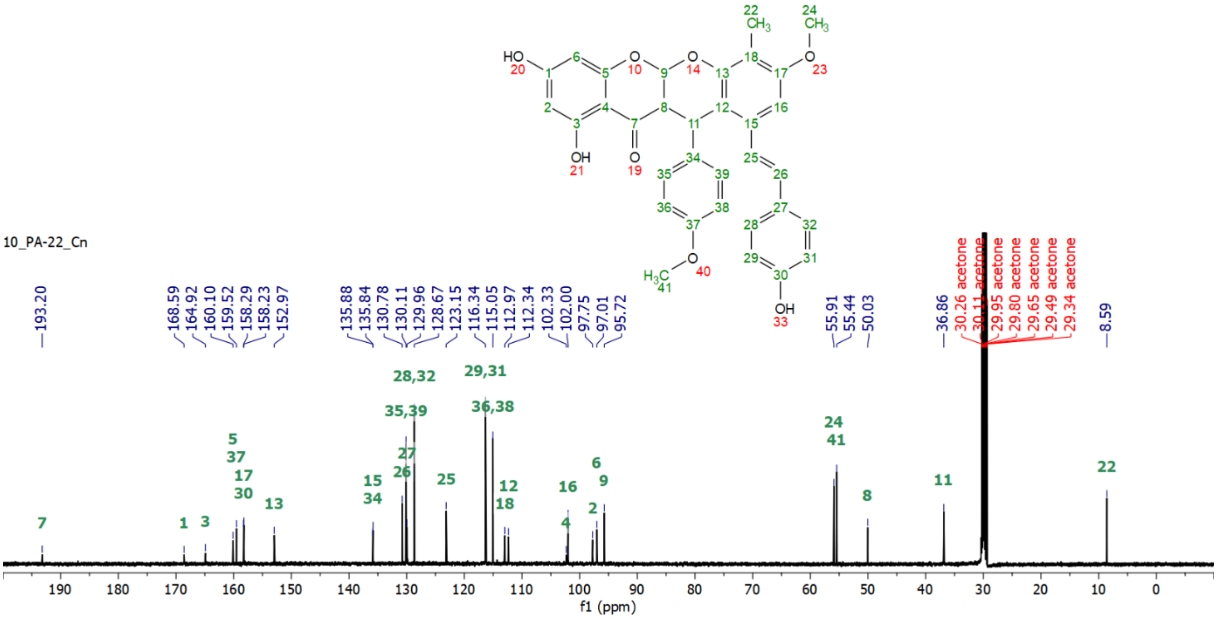

Figure S33. HSQC spectrum of prosperin C (**10**)

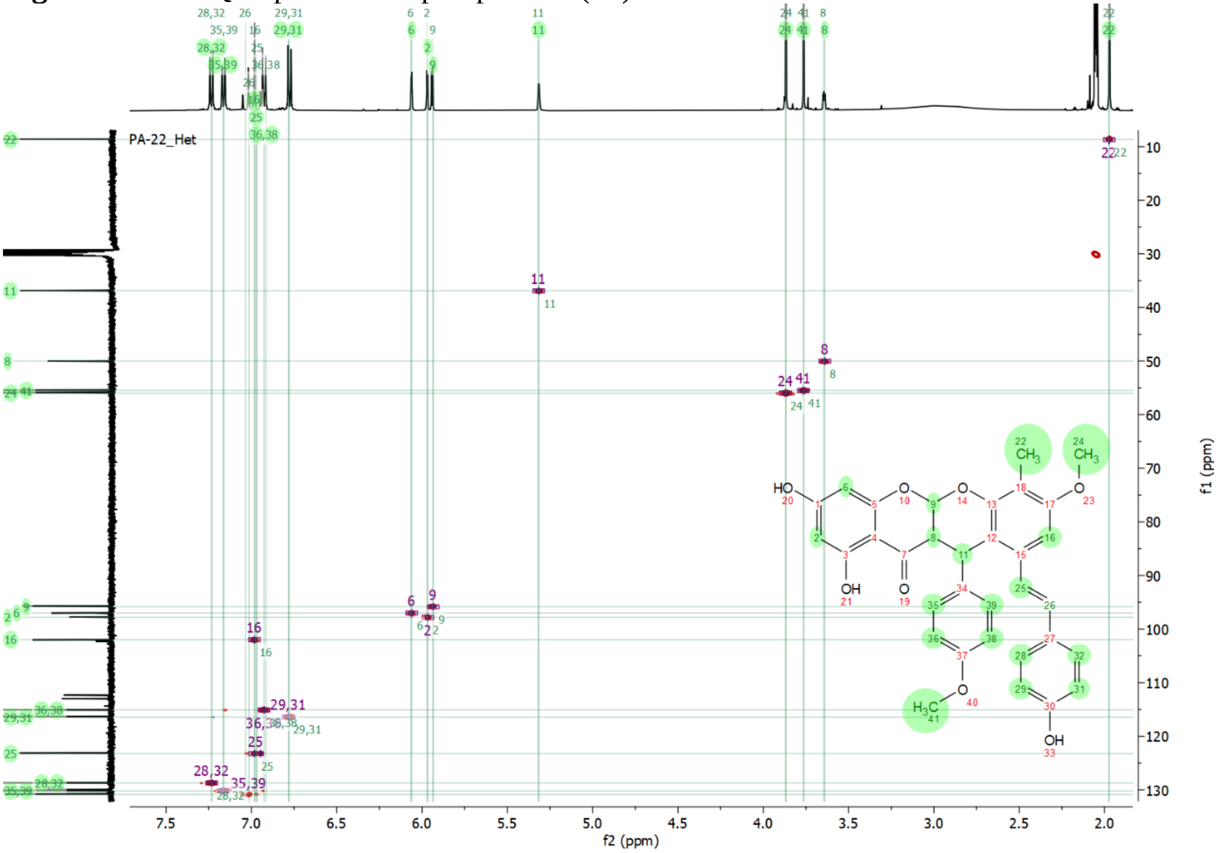

Figure S34. COSY spectrum of prosperin C (**10**)

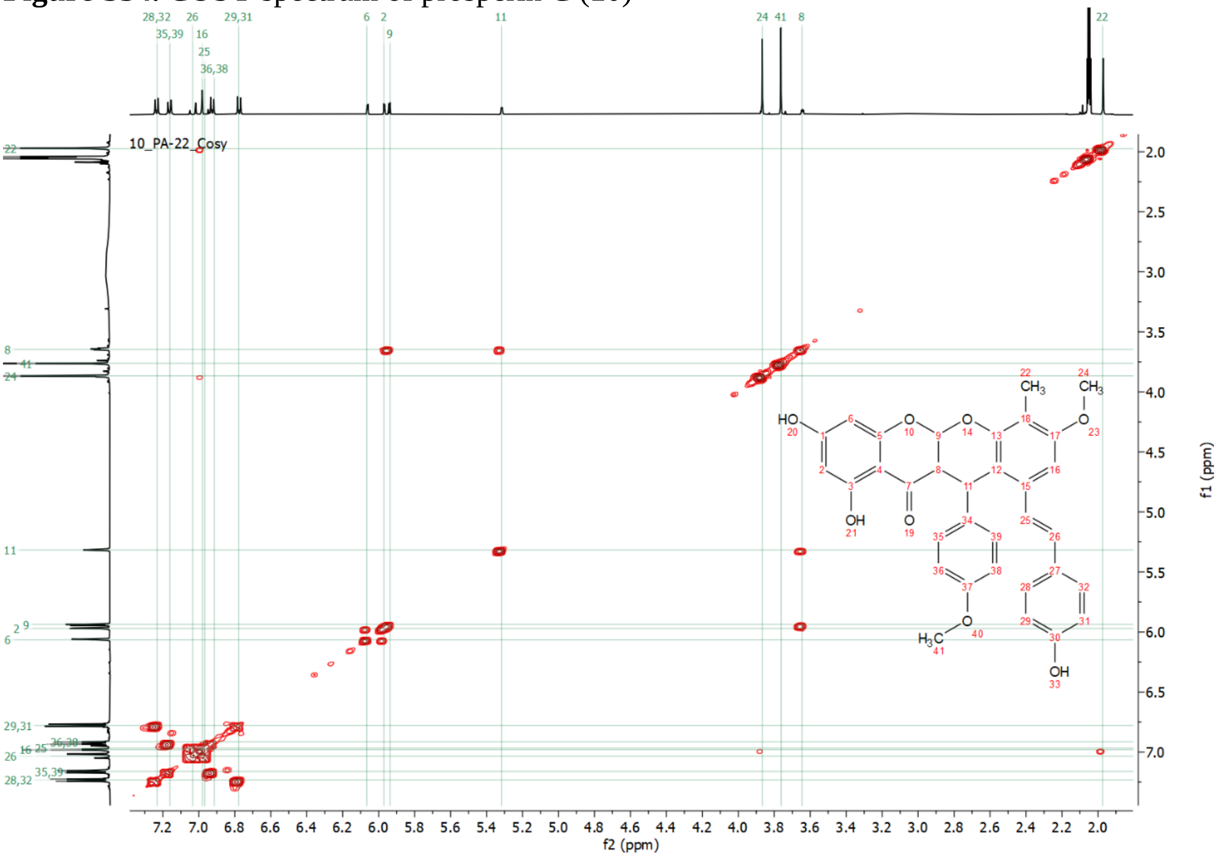

Figure S35. H2BC spectrum of prosperin C (**10**)

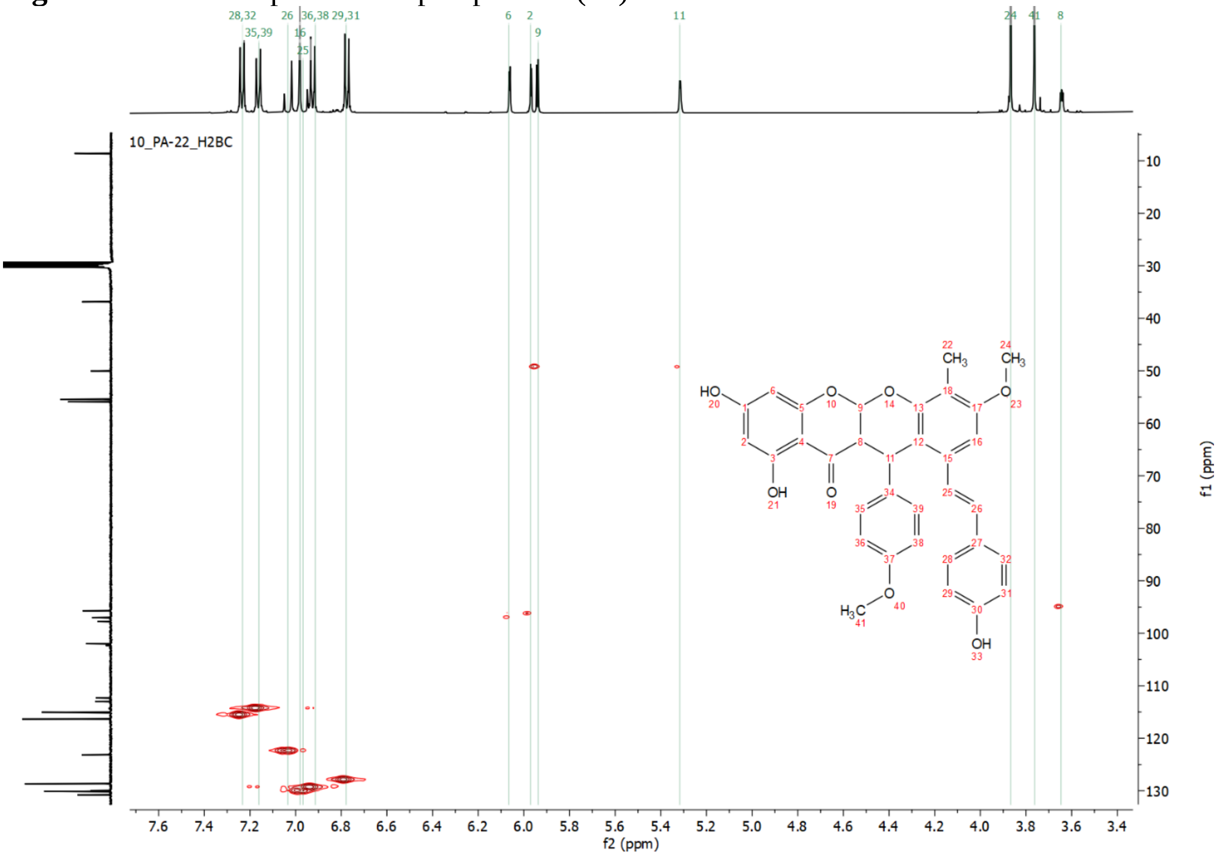

Figure S36. HMBC spectrum of prosperin C (**10**)

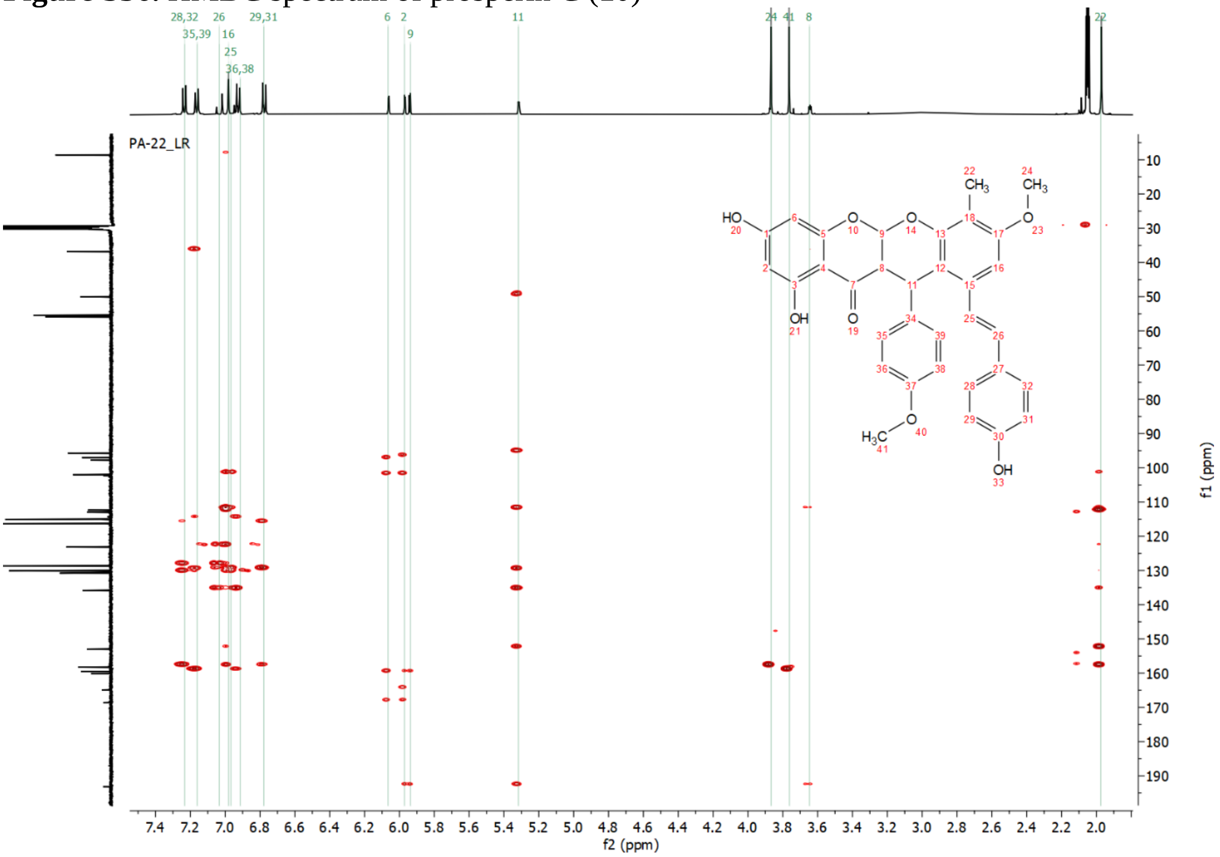

Figure S37. NOESY spectrum of prosperin C (**10**)

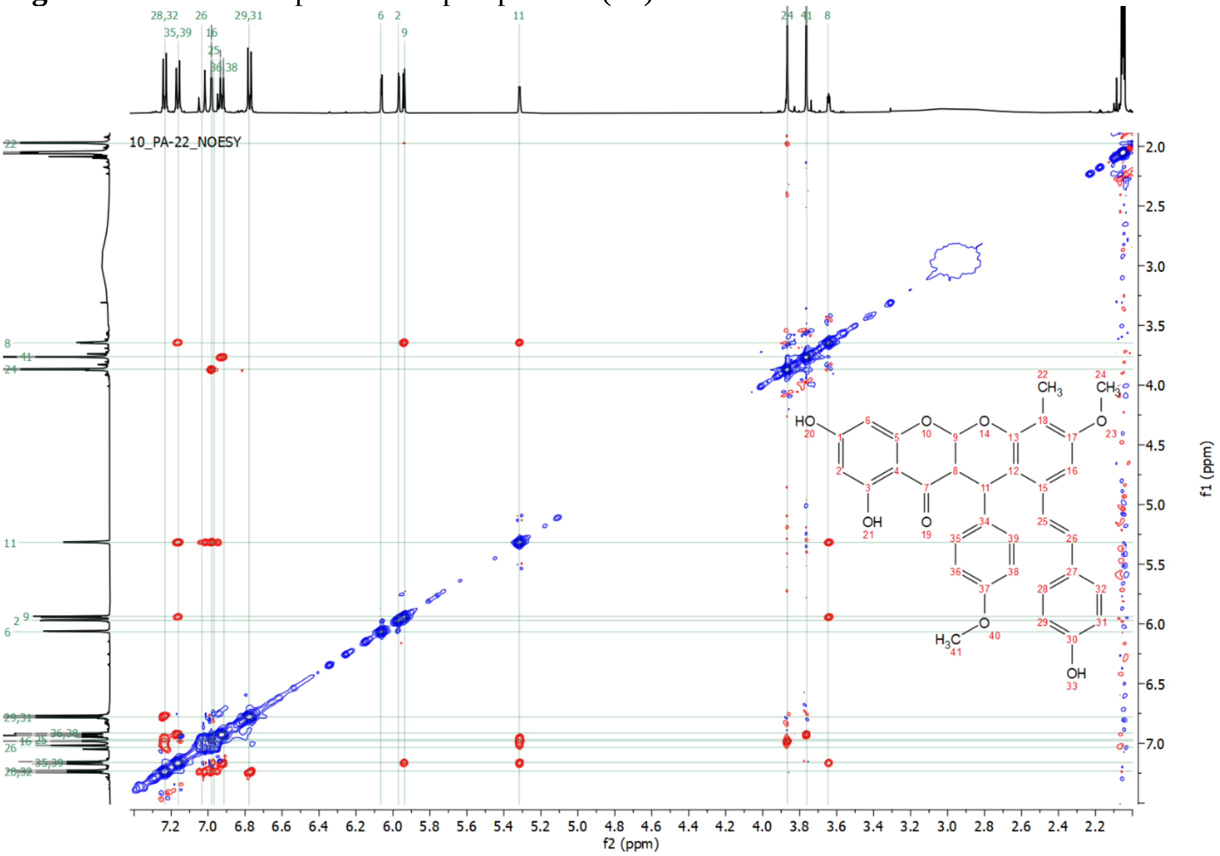

Figure S38. ECD spectrum of prosperin C (**10**) in MeOH

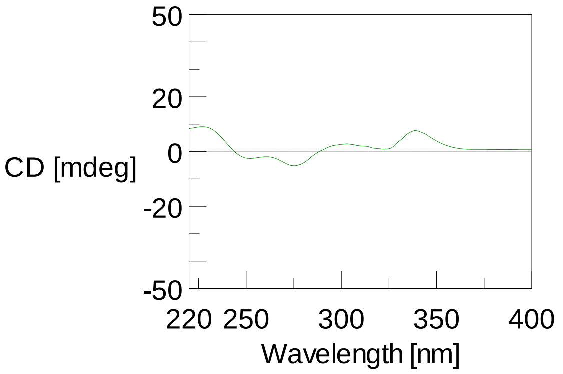

ECD (c 0.2 mg/ml, MeOH)

$\Delta\varepsilon_{227} = +7.56$ $[\theta]_{227} = +24958$

$\Delta\varepsilon_{252} = -2.13$ $[\theta]_{252} = -7012$

$\Delta\varepsilon_{275} = -4.34$ $[\theta]_{275} = -14301$

$\Delta\varepsilon_{339} = +6.38$ $[\theta]_{339} = +21038$

Figure S39. IR spectrum of prosperin C (**10**)

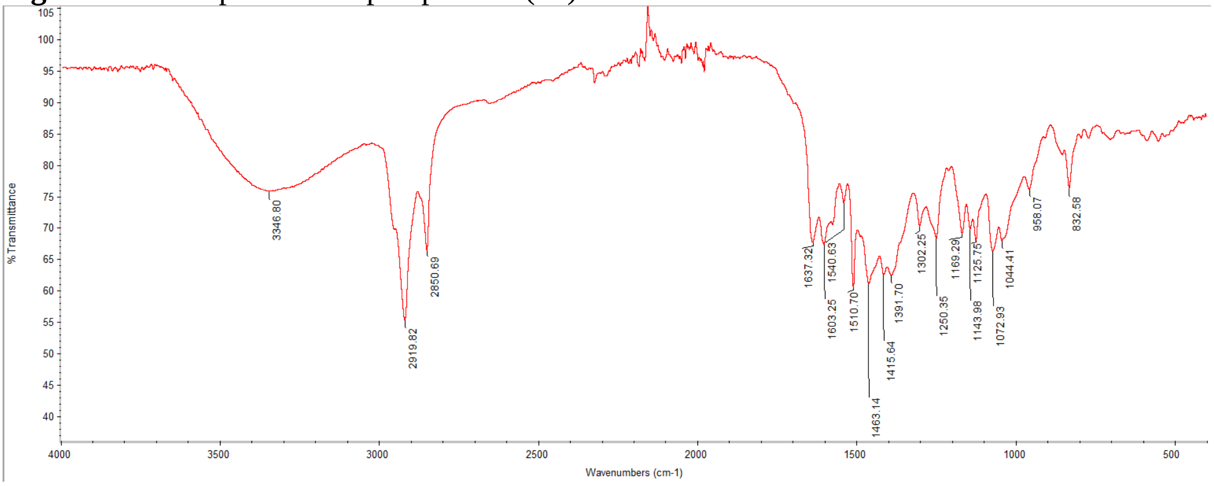

Figure S40. UV spectrum of prosperin C (**10**)

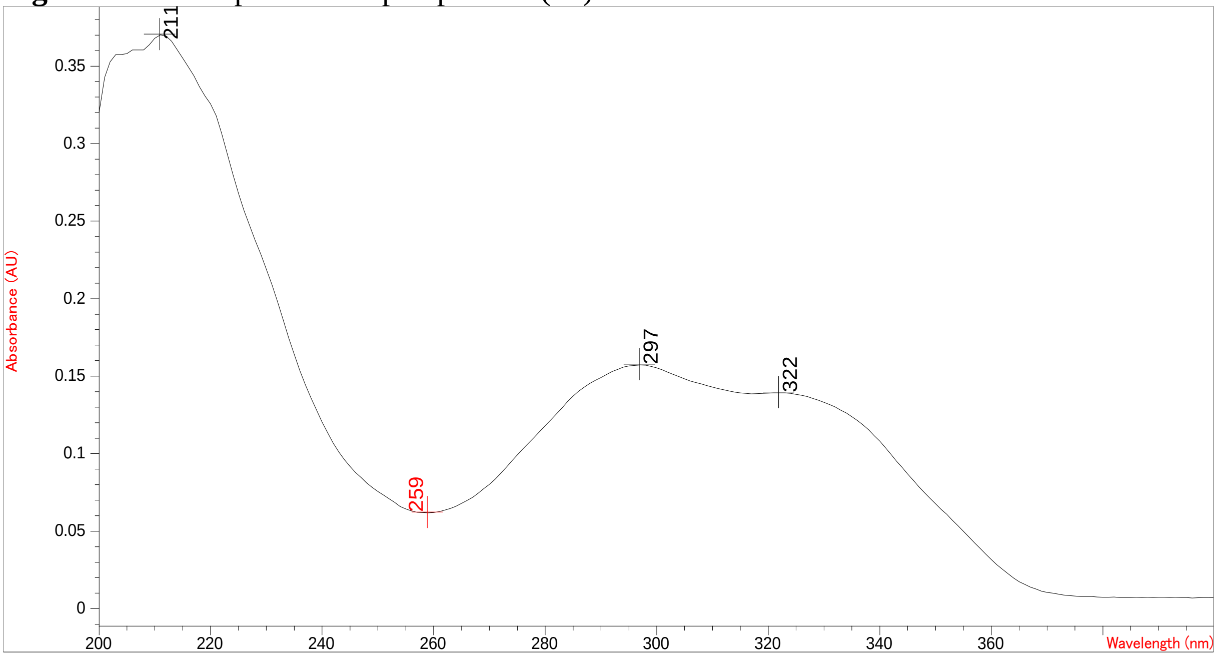

Details about computational NMR calculations of prosperin A (8) in chloroform

In addition to modeling prosperin A in actone (see main text), we calculated the NMR shifts in chloroform as well. The conformer library was generated manually resulting in 24 confomers for both form A and B. Geometry optimizations done using the same level of theory used in the main text, but NMR calulcations were done at the mPW1PW91/6-311+G(2d,p) scrf=(smd,solvent=chloroform) level of theory. Conformers for structure A ranged from 0 to 4.12 kcal/mol while structure B had conformers ranging from 0 to 3.80 kcal/mol. Comparison between the hydrogen and carbon NMR are shown for the highlighted hydrogens and carbons are below.

Table S2. Experimental and computed ^{1}H NMR of prosperin A (**8**) in $CDCl_3$

	Me	a	b	c	d	f	e	g
Measured	4.14	7.11	6.79	4.34	4.19	3.15	2.81-2.87	2.75
**A (computed)**	4.13	7.20	6.77	**4.12**	4.02	**3.33**	2.77	**2.53**
**B (computed)**	**3.75**	7.20	6.74	4.19	**3.91**	3.03	2.86	**3.00**

Difference from the measured values: **red** > 0.30 ppm, **blue** 0.20 ~ 0.30 ppm

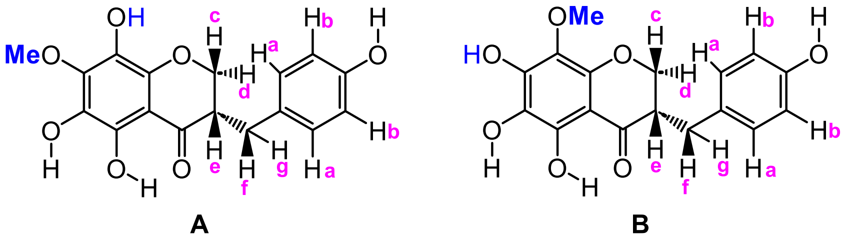

Table S3. Experimental and computed ^{13}C NMR of prosperin A (**8**) in $CDCl_3$

	1	2	3	4	5	6	7	8
Measured	199.1	154.4	130.4	115.6	69.6	61.1	47.7	32.0
**A (computed)**	199.1	155.3	130.7	113.9	68.9	58.8	50.0	32.7
**B (computed)**	197.6	155.3	130.5	113.8	69.3	58.7	49.3	32.4

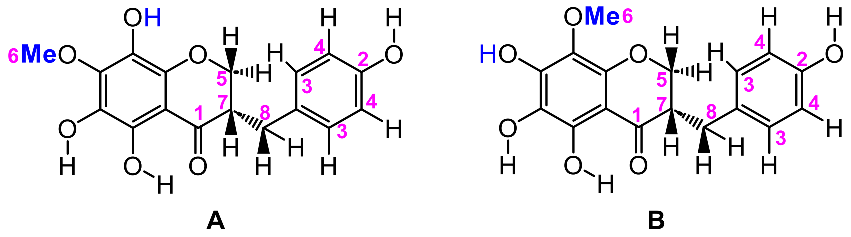

Table S4. Comparison of ^{1}H and ^{13}C NMR of prosperin B (**9**) in CD_3COCD_3

**no.**	δ_C, type (measured)	δ_C (calculated)	δ_H, mult. (J in Hz) (measured)	δ_H (calculated)
2	157.3, CH	158.2	7.78, d (1.1)	8.12
3	125.1, C	123.8		
4	182.2, C	179.5		
4a	105.6, C	102.2		
5	163.5, C	162.0		
6	99.8, CH	98.3	6.25, d (2.1)	6.16
7	165.1, C	162.0		
8	94.4, CH	93.0	6.38, d (2.1)	6.42
8a	159.12, C	157.1		
9	35.9, CH	38.7	5.97, bs	5.64
10	133.2, C	129.8		
11/15	130.2, CH	127.5	7.24–7.21, m	7.09
12/14	114.3, CH	112.1	6.86–6.83, m	6.79
13	159.08, C	157.6		

5-OH	-		12.76, s	-
7-OH	-		10.00–9.00, bs	-
13-OCH_3	55.4, CH_3	53.0	3.76, s	3.64
2′	68.1, CH_2	67.6	5.38, d (1.8)	5.20
3′	128.1, C	127.1		
4′	185.9, C	181.6		
4a′	103.2, C	102.2		
5′	164.2, C	163.9		
6′	109.6, C	107.9		
7′	165.9, C	164.6		
8′	96.1, CH	96.3	5.99, s	5.89
8a′	161.8, C	160.5		
9′	137.5, C	138.3	7.75–7.73, m	7.89
5′-OH	-		13.62, s	-
7′-OH	-		10.00–9.00, bs	-
1″	126.7, C	126.2		
2″/6″	133.5, CH	133.4	7.36–7.33, m	7.29

^{13}C CMAD: 1.6; ^{1}H CMAD: 0.2

Figure S41. ECD calculations for prosperin B (**9**) with experimental values (green) and computed *S* and *R* enantiomers (blue and red respectively)

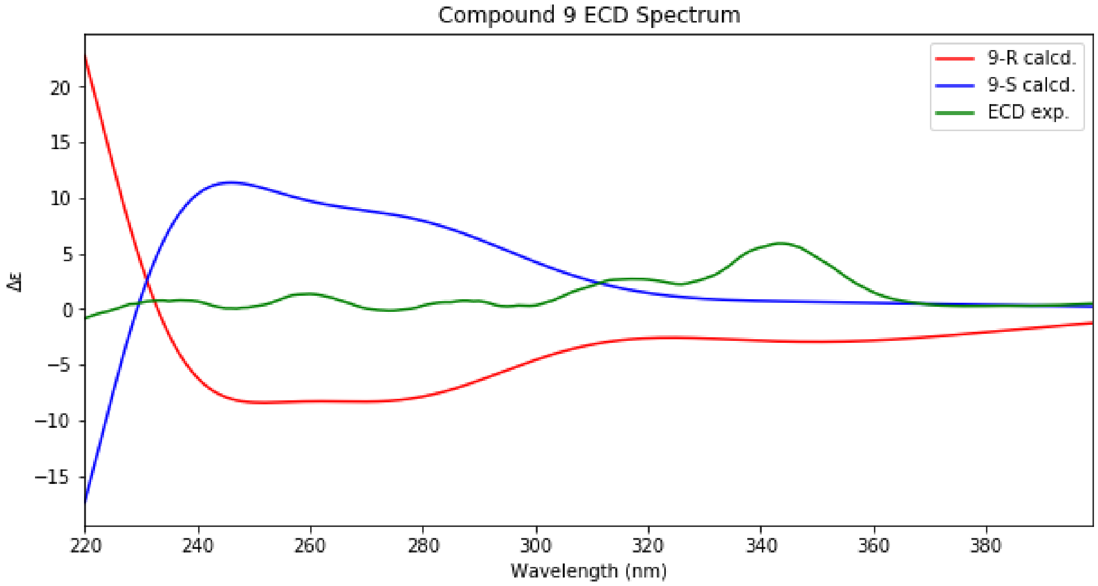

Figure S42. ECD calculations for alternative prosperin C (**10**) stereoisomers

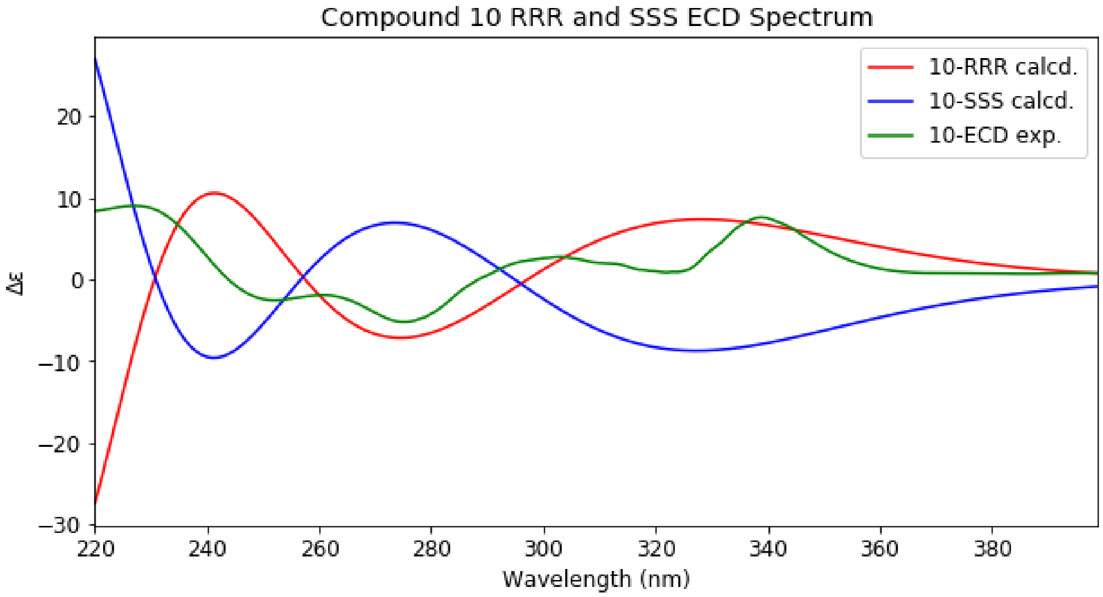

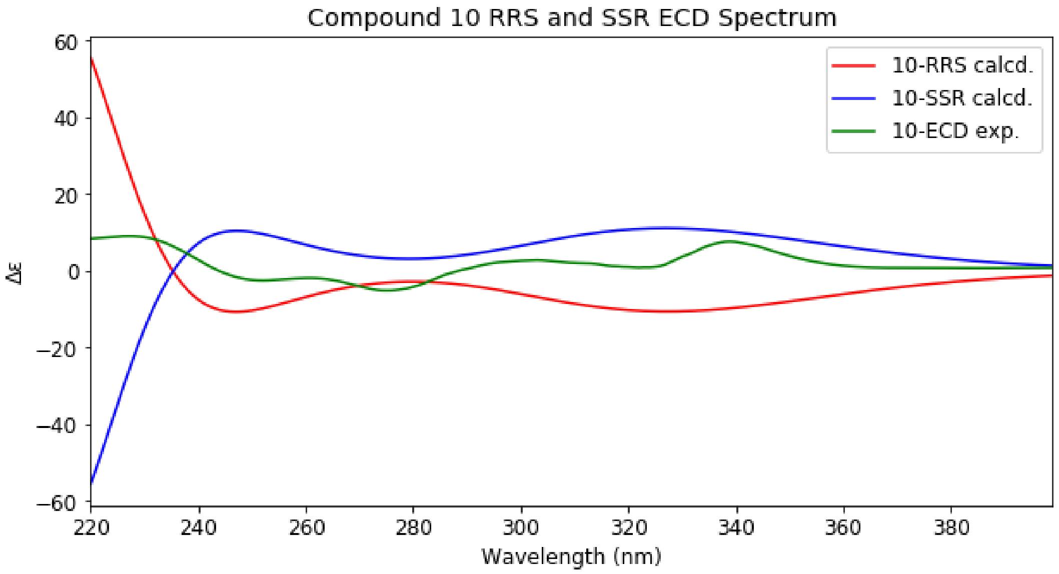
Compound 10 RRS and SSR ECD Spectrum
10-RRS calcd.
10-SSR calcd.
10-ECD exp.
Δε
Wavelength (nm)
60
40
20
0
−20
−40
−60
220
240
260
280
300
320
340
360
380

Figure S43. NMR calculations for alternative prosperin C (**10**) stereoisomers

			**10**			
**no.**	δ_C, type, exp.	δ_C, Computed as (2S,3R,9S)	δ_C, Computed as (2S,3R,9R)	δ_H, mult. (*J* in Hz), exp.	δ_H Computed as (2S,3R,9S)	δ_H Computed as (2*S*,3*R*,9*R*)
2	95.7, CH	94.3	96.3	5.94, d (3.6)	5.9	6.0
3	50.0, CH	51.5	49.0	3.64, dd (3.6, 2.3)	3.4	3.3
4	193.2, C	192.2	196.2			
4a	102.3, C	102.2	103.7			
5	164.9, C	163.9	163.6			
6	97.8, CH	95.1	94.5	5.97, d (2.1)	6.0	5.8
7	168.6, C	164.6	164.1			
8	97.0, CH	94.4	93.5	6.06, d (2.1)	6.1	5.7
8a	160.1, C	159.4	160.1			
9	36.9, CH	38.8	40.1	**5.32, d (2.3)**	**5.4**	**4.8**
10	135.8, C	137.0	132.4			
11/15	130.1, CH	129.8	131.2	7.20–7.16, m	7.2	7.1

12/14	115.1, CH	112.5	111.7	6.96–6.92, m	6.9	6.7
13	159.5, C	158.1	158.1			
5-OH	-			11.79, s	11.6	11.7
13-OCH_3	55.4, CH_3	52.9	52.7	3.76, s	3.7	3.5
1′	135.9, C	136.0	136.0			
2′	112.3, C	111.3	113.6			
3′	153.0, C	152.1	149.9			
4′	113.0, C	113.1	113.4			
5′	158.3, C	156.7	155.9			
6′	102.0, CH	99.8	100.8	6.98, s, overlap	6.8	6.9
7′	123.2, CH	124.3	123.3	6.96, d (16.1), overlap	7.2	7.1
8′	130.8, CH	130.5	130.7	7.03 d (16.1)	7.0	6.9
4′-CH_3	8.6, CH_3	8.4	8.8	1.97, s	2.0	2.3

5′-OCH$_3$	55.9, CH_3	53.0	53.0	3.87, s	3.8	3.8
1″	130.0, C	129.0	128.9			
2″/6″	128.7, CH	128.4	128.5	7.26–7.22, m	7.4	7.4
3″/5″	116.3, CH	113.9	113.9	6.80–6.76, m	6.9	6.9
4″	158.2, C	156.2	156.2			
CMAD		1.4	1.7		0.10	0.18
largest outlier		Δδ 4.0	Δδ 4.5		Δδ 0.24	Δδ 0.54
